# Multidimensional endotyping in patients with severe asthma reveals inflammatory heterogeneity in matrix metalloproteinases and chitinase 3–like protein 1

**DOI:** 10.1016/j.jaci.2015.11.020

**Published:** 2016-07

**Authors:** Timothy S.C. Hinks, Tom Brown, Laurie C.K. Lau, Hitasha Rupani, Clair Barber, Scott Elliott, Jon A. Ward, Junya Ono, Shoichiro Ohta, Kenji Izuhara, Ratko Djukanović, Ramesh J. Kurukulaaratchy, Anoop Chauhan, Peter H. Howarth

**Affiliations:** a Clinical and Experimental Sciences, University of Southampton Faculty of Medicine, Sir Henry Wellcome Laboratories, Southampton University Hospital, Southampton, United Kingdom; b NIHR Southampton Respiratory Biomedical Research Unit, Southampton University Hospital, Southampton, United Kingdom; c Portsmouth Hospitals NHS Trust, Portsmouth, United Kingdom; d Shino-Test Corporation, Kanagawa, Japan; e Department of Laboratory Medicine, Saga Medical School, Saga, Japan; f Department of Biomolecular Sciences, Saga Medical School, Saga, Japan; g Department of Respiratory Medicine, Southampton General Hospital, Southampton, United Kingdom

**Keywords:** Asthma, cytokines, eosinophils, neutrophils, phenotype, endotype, heterogeneity, matrix metalloproteinase, chitinase 3–like protein 1, topological data analysis, ACQ, Asthma Control Questionnaire, ECP, Eosinophil cationic protein, Feno, Fraction of exhaled nitric oxide, FGF, Fibroblast growth factor, GINA, Global Initiative for Asthma, HAD, Hospital Anxiety and Depression, ICS, Inhaled corticosteroid, K-S, Kolmogorov-Smirnov, MMP, Matrix metalloproteinase, TDA, Topological data analysis, YKL-40, Chitinase 3–like protein 1

## Abstract

**Background:**

Disease heterogeneity in patients with severe asthma and its relationship to inflammatory mechanisms remain poorly understood.

**Objective:**

We aimed to identify and replicate clinicopathologic endotypes based on analysis of blood and sputum parameters in asthmatic patients.

**Methods:**

One hundred ninety-four asthmatic patients and 21 control subjects recruited from 2 separate centers underwent detailed clinical assessment, sputum induction, and phlebotomy. One hundred three clinical, physiologic, and inflammatory parameters were analyzed by using topological data analysis and Bayesian network analysis.

**Results:**

Severe asthma was associated with anxiety and depression, obesity, sinonasal symptoms, decreased quality of life, and inflammatory changes, including increased sputum chitinase 3–like protein 1 (YKL-40) and matrix metalloproteinase (MMP) 1, 3, 8, and 12 levels. Topological data analysis identified 6 clinicopathobiologic clusters replicated in both geographic cohorts: young, mild paucigranulocytic; older, sinonasal disease; obese, high MMP levels; steroid resistant T_H_2 mediated, eosinophilic; mixed granulocytic with severe obstruction; and neutrophilic, low periostin levels, severe obstruction. Sputum IL-5 levels were increased in patients with severe particularly eosinophilic forms, whereas IL-13 was suppressed and IL-17 levels did not differ between clusters. Bayesian network analysis separated clinical features from intricately connected inflammatory pathways. YKL-40 levels strongly correlated with neutrophilic asthma and levels of myeloperoxidase, IL-8, IL-6, and IL-6 soluble receptor. MMP1, MMP3, MMP8, and MMP12 levels were associated with severe asthma and were correlated positively with sputum IL-5 levels but negatively with IL-13 levels.

**Conclusion:**

In 2 distinct cohorts we have identified and replicated 6 clinicopathobiologic clusters based on blood and induced sputum measures. Our data underline a disconnect between clinical features and underlying inflammation, suggest IL-5 production is relatively steroid insensitive, and highlight the expression of YKL-40 in patients with neutrophilic inflammation and the expression of MMPs in patients with severe asthma.

Asthma is a chronic inflammatory disorder of the airways characterized by variable airflow obstruction and airway remodeling and mediated by a variety of inflammatory mediators and cells, including mast cells, T cells, eosinophils, and neutrophils.^1^ There is now recognition of considerable disease heterogeneity within the spectrum of clinical asthma, the precise nature of which remains to be defined, and this currently constitutes a significant barrier to research.^2^ It is postulated that distinct subgroups of asthma exist, which have been termed endotypes, meaning “a subtype of a condition defined by distinct pathophysiological mechanisms.”^3^ A better understanding of such endotypes and their relationship to distinct underlying disease mechanisms should enable identification of novel therapeutic targets and facilitate the aim of stratified medicine (ie, the efficient targeting of specific therapies to subgroups of subjects likely to benefit most).

To date, several groups have reported cluster analyses of patient cohorts to investigate possible disease endotypes.^4^, ^5^, ^6^, ^7^, ^8^, ^9^ However, these are often limited by a lack of robust statistical validation in replication cohorts or have generated clusters the identity of which is dominated by predominantly clinical parameters, such as pulmonary physiology or participants' demographics, without providing significant insight into the underlying pathophysiology. Furthermore, techniques like principle component analysis tend to accentuate separation between clusters, which might in reality represent groupings within a continuum of disease rather than clear-cut entities.^9^ Recently, we have piloted a new analytic approach to such large clinical data sets by using network analyses that allow truly multidimensional analysis of clusters and provide visual representations of the data that reveal continuities within data sets.^10^

The aim of this study was to identify and independently replicate distinct multidimensional clinicopathobiologic clusters of severe asthma from the participants in the Wessex Severe Asthma cohort who had induced sputum and peripheral blood biomarker measures, as well as detailed clinical characterization. We aimed to cluster participants using only parameters that could be available to a clinician in tertiary care with access to sputum induction facilities and then to investigate the disease mechanisms of airway inflammation in each of these clusters by using more advanced immunologic assays.

## Methods

### Southampton participants (derivation cohort)

**Fig E1:**
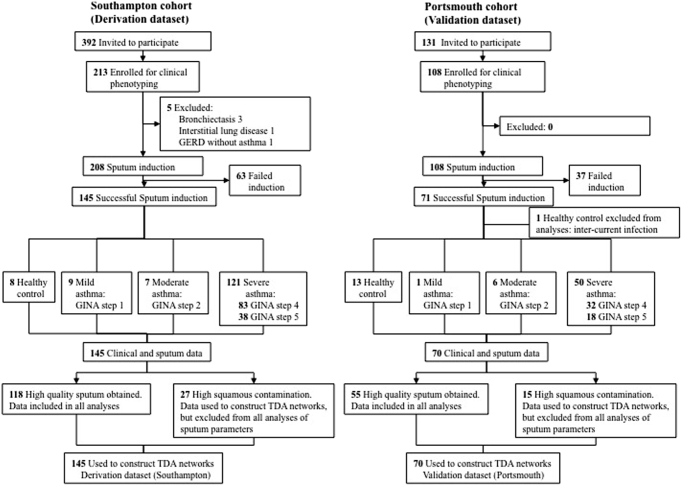


The derivation cohort comprised 213 adult participants (18-70 years) enrolled for clinical phenotyping in the Wessex Severe Asthma Cohort, at the NIHR Southampton Respiratory Biomedical Research Unit. Five were excluded because of alternative diagnoses of bronchiectasis (n = 3), interstitial lung disease (n = 1), and gastroesophageal reflux without asthma. One hundred forty-five participants underwent successful sputum induction, with the emphasis on severe asthma (n = 121) and inclusion of 8 healthy nonatopic participants, 9 patients with mild asthma receiving β_2_-agonists alone, and 7 patients with moderate asthma receiving inhaled corticosteroids (ICSs). Thirty-eight of the 121 patients with severe asthma with persistent symptoms despite high-dose ICSs and other therapy were also receiving daily oral corticosteroids (Table I and see the Methods section and Fig E1 in this article's Online Repository at www.jacionline.org).

### Portsmouth participants (validation cohort)

The validation cohort comprised 108 adult participants (18-70 years) enrolled by a separate study team from outpatient clinics at Queen Alexandra Hospital, Portsmouth. Seventy-one participants underwent successful sputum induction: 13 healthy nonatopic participants, 1 patient with mild asthma, 6 patients with moderate asthma, and 50 patients with severe asthma with persistent symptoms despite high-dose ICSs (n = 32) and oral corticosteroids (n = 18, Table II and see Fig E1).

### Study procedures

Participants were assessed based on history; examination; questionnaires, including the Asthma Control Questionnaire (ACQ),^11^ Asthma Quality of Life Questionnaire,^12^ Hospital Anxiety and Depression (HAD) Scale,^13^ Sino-Nasal Outcome Test 20,^14^ and Short-Form 36 Health Survey^15^; skin prick tests with common aeroallergens; spirometry with albuterol reversibility; exhaled nitric oxide measurements; the University of Pennsylvania smell identification test^16^; and serum IgE and urinary cotinine measurements. Sputum samples were obtained by means of hypertonic saline induction and processed as previously described.^17^ Fifty-five different inflammatory mediators were measured in serum and sputum by using ELISAs or cytokine bead arrays (see the Methods section in this article's Online Repository).

The study was approved by the Southampton and South West Hampshire Research Ethics Committee A (09/H0502/37). All participants provided informed consent.

### Statistical analysis

Data were analyzed initially by using topological data analysis (TDA) to define multidimensional clusters in the derivation and validation cohorts separately. Standard statistical methods were then applied to define the features of these clusters. In a separate analysis to define relationships between these parameters, Bayesian network analysis was then applied to all the pathobiologic and clinical features on the highest quality data from both cohorts combined.

Data are expressed as medians with interquartile ranges, unless stated otherwise. Data were logarithmically transformed if they were not normally distributed. For all analyses, 2-tailed *P* values of less than .05 were considered significant. Data were compared between the healthy and control groups by using Mann-Whitney *U* or Student *t* tests and between each asthma severity group and control subjects by using the Kruskal-Wallis test or ANOVA, depending on data distribution. For the latter, an overall 5% significance level was adjusted for multiple comparisons by using the Bonferroni method. Correlations were tested with the Spearman *r* statistic. Kolmogorov-Smirnov (K-S) tests identified significant differences between distributions within a single cluster. Data were analyzed with Prism 6.0 (GraphPad Software, San Diego, Calif) and SPSS 21.0 (IBM, Armonk, NY) software.

Network analyses (TDA and Bayesian network analysis) were performed, as previously described.^10^ Networks were generated from all participants with the most complete data (n = 145 for the derivation data set and n = 70 for the validation data set) after missing data (6.1% of data set) were imputed by using the mean of 5 multiple imputations. Subsequent analyses of sputum parameters used only data from the highest quality sputum samples (n = 118 for the derivation data set and n = 55 for the validation data set) and without imputation. Terms used to generate the networks are described in Tables E1 and E2 in this article's Online Repository at www.jacionline.org.

### TDA

To identify multidimensional features within the data sets, which might not be apparent by using traditional methods, we used TDA. This is particularly suited to complex biological data sets, representing a high-dimensional data set as a structured 3-dimensional network. Each node comprises participants similar to each other in multiple dimensions. Edges connect nodes that contain shared data points. Statistical tests can then be performed on groups or features that emerge from the inherent structure of the data set. This technique provides a geometric representation of the data,^18^, ^19^ is independent of prior hypotheses, and detects multidimensional features within the data that become apparent on visualization. As a consequence, topological networks capture interesting structure, even in very small data sets.

TDA was performed, as previously described,^10^, ^19^ by using Ayasdi Core 1.59 (Ayasdi, Menlo Park, Calif), constructing networks with the 29 parameters listed in Table E1. Variance-normalized Euclidean distance was used as a distance metric with 2 filter functions: principal and secondary metric singular value decomposition. Resolution was set at 30 and gain at 3 (derivation) or 4 (validation) and selected to provide network structures that permitted identification of subgroups. K-S tests identified parameters that differentiated each cluster from the rest of the structure. Comparisons between multiple clusters used 1-way ANOVA, with *post hoc* tests with the Bonferroni correction.

### Bayesian network analysis

Interconnectivity between clinical and pathobiologic parameters was explored by using Bayesian network analysis (Genie 2.0; Decision Systems Laboratory, University of Pittsburgh, Pittsburgh, Pa). Data were discretized to describe nonlinear correlations into 2 (binary variables) or 4 or 5 (continuous variables) bins. Seventy-four parameters were included in analyses (see Table E2) on the 173 participants (including 17 healthy control subjects) from both cohorts with the highest quality sputum data and without imputation. The strengths of associations found to be significant in this analysis were analyzed by using Spearman correlations.

## Results

**Fig 1:**
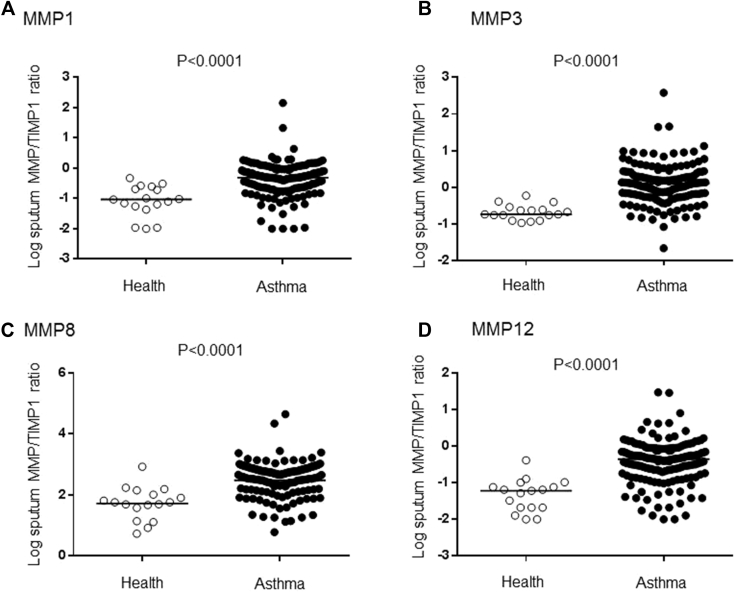
Protease/antiprotease balance in asthmatic patients. MMP/tissue inhibitor of metalloproteinases *(TIMP-1)* ratios in sputum in asthmatic patients compared with healthy subjects for MMP1 **(A)**, MMP3 **(B)**, MMP8 **(C)**, and MMP12 **(D)** are shown. *Horizontal lines* show medians. Statistical comparisons were done with Student *t* tests on log-transformed data.

First, we investigated which of the 103 clinical, physiologic, and pathobiologic parameters measured were associated with severe asthma (Global Initiative for Asthma [GINA] step 4 and 5). Features that differed significantly in K-S tests between patients with severe asthma and healthy subjects in both the derivation data set (n = 145 participants) and the validation data set (n = 70) are presented in Table III. The presence of severe asthma was associated with symptoms of anxiety and depression or nasal dysfunction, decreased quality-of-life scores, obesity, obstructive spirometry, and increased reversibility. Pathobiologic parameters associated with a diagnosis of severe asthma were neutrophilic sputum; an increase in peripheral blood neutrophil counts; serum and sputum chitinase 3–like protein 1 (YKL-40) levels; sputum matrix metalloproteinase (MMP) 1, MMP3, MMP8, and MMP12 levels (*P* < .0001 each, Fig 1); vascular endothelial growth factor, IL-5, IL-6, IL-8, and IL-6 soluble receptor levels; and a decrease in sputum macrophage counts and levels of tissue inhibitor of metalloproteinases 1, fibroblast growth factor, IL-1 receptor antagonist, and IL-2.

### TDA to identify clusters

**Fig E2:**
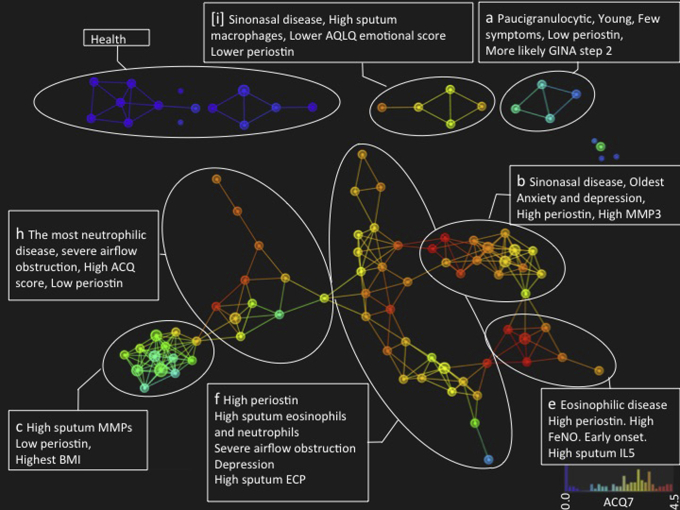


**Fig E3:**
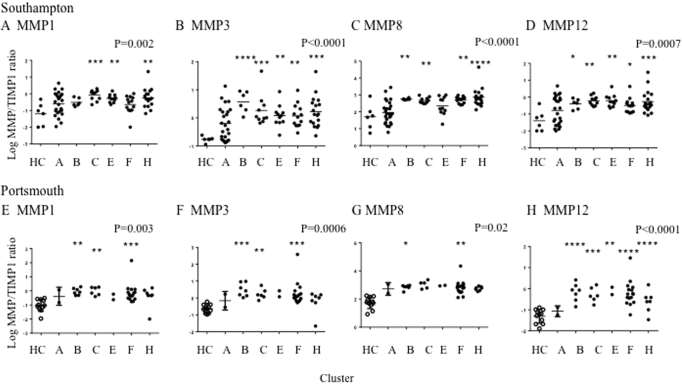


**Fig E4:**
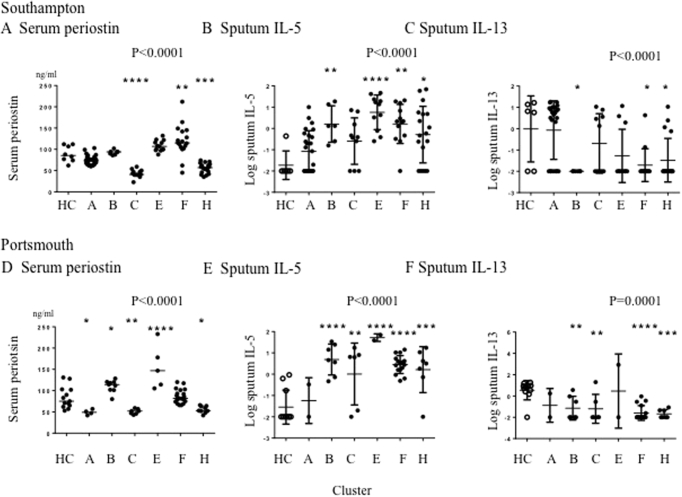


**Fig 2:**
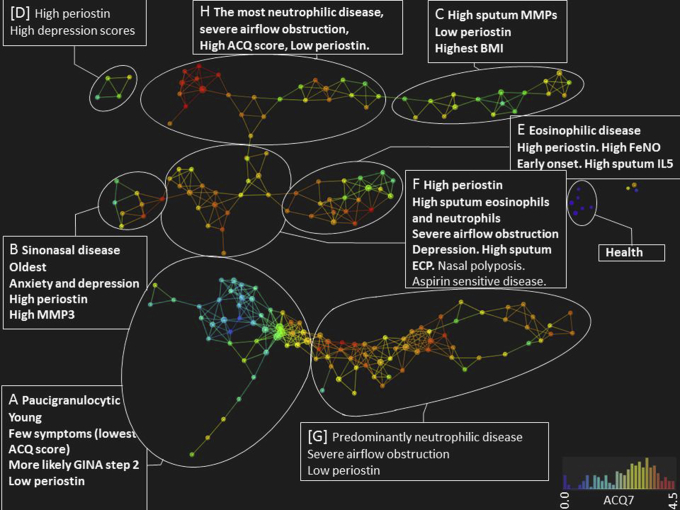
Multidimensional clinicopathological clusters in asthmatic patients in the derivation data set (Southampton cohort). A topological network generated by using 22 clinical and pathological features together identifies 1 healthy (in *blue*) and 8 distinct clinicopathobiologic asthma clusters **(A-H)**. The network is colored according to ACQ7 scores, with the most symptomatic subjects in *red*. The TDA used 145 subjects with the most complete data: *metric*, variance-normalized Euclidean; *lenses*, principal and secondary singular value decomposition (resolution, 30; gain, 3.0/3.0×, equalized) and presence/absence of asthma; *node size*, proportional to the number of subjects in the node. Color bars: *red*, highest ACQ7 score; *blue*, healthy participants. Features in boldface were replicated in the validation data set. *GINA*, Global Initiative for Asthma.

Next, we applied TDA to the Southampton cohort (derivation) data sets to identify multidimensional clinicopathobiologic clusters. The network was generated by using only 29 clinical, physiologic, and cellular parameters (see Table E1) with the potential to be available to a tertiary care clinician. Subsequent cluster analyses were then performed on data available from all 103 parameters. Eight clusters of asthmatic patients (A-H) were identified, as described in Tables I and IV and Fig 2. Of these, 6 clusters (A-C, E, F, and H) were subsequently replicated when the same analysis was applied to the geographically distinct Portsmouth (validation) cohort (Tables II and IV and see Figs E2-E4 in this article's Online Repository at www.jacionline.org), which also identified a small additional cluster (cluster i) not present in the Southampton cohort. Healthy control subjects formed distinct clusters in both analyses.

Of the 6 clusters replicated in both data sets, cluster A (young, mild, paucigranulocytic) comprises participants with predominantly paucigranulocytic sputum, few symptoms (the lowest ACQ7 scores, 0.8-1.6), and low serum periostin levels who are young (lowest median ages, 34-38 years) and more likely to be at GINA treatment step 2 (low-dose maintenance ICS).

Subjects in cluster B (older, sinonasal disease) have the highest median age, more symptoms of anxiety and depression (highest median HAD score, 12-27), more nasal symptoms (highest Sino-Nasal Outcome Test 20 score), and high levels of serum periostin and sputum MMP3.

Subjects in cluster C (obese, high MMP levels) have the highest body mass index (30.9-36.4 kg/m^2^); increased sputum MMP1, MMP2, and MMP8 concentrations; and low serum periostin levels.

Subjects in cluster E (steroid-resistant T_H_2-mediated, eosinophilic) have high serum periostin levels, sputum eosinophilia, sputum IL-5 levels, and fraction of exhaled nitric oxide (Feno) levels despite high-dose ICSs (1600-2000 μg of beclomethasone dipropionate) or oral corticosteroids (40% to 46% of participants).

Subjects in cluster F (mixed granulocytic inflammation with severe obstruction) have both sputum eosinophilia and neutrophilia with lower prebronchodilator FEV_1_ values and FEV_1_/forced vital capacity ratios associated with higher sputum periostin and eosinophil cationic protein (ECP) levels and high HAD scores.

Subjects in cluster H (neutrophilic disease with severe obstruction and low periostin levels) have high sputum neutrophil counts with fixed airflow obstruction (low prebronchodilator and postbronchodilator FEV_1_) associated with very high symptom scores (median ACQ7, 3.3-3.4) and low serum periostin levels.

Of the clusters that were not replicated in both data sets, both clusters D and i were small (n = 4 and 5, respectively) and therefore might represent model overfitting. Lastly, cluster G shared many features with cluster H, comprising a second large cluster of participants with blood and sputum neutrophilia, high symptom scores, and low serum periostin levels. When clusters G and H were compared directly, cluster G had higher prebronchodilator and postbronchodilator FEV_1_, higher sputum macrophage counts, and higher serum periostin levels and were less neutrophilic, with lower sputum neutrophil counts and sputum myeloperoxidase (MPO), MMP8, and MMP9 levels (data not shown). Thus clusters G and H could be considered to represent milder and more severe subgroups, respectively, of neutrophilic asthma with low periostin levels.

Features of specific interest were compared across these TDA clusters. Serum periostin levels were significantly lower in clusters C and H in both the training and validation cohorts (see Fig E4). Although sputum IL-5 concentrations were significantly increased in severe clusters B through H, sputum IL-13 concentrations were significantly decreased in most of the severe clusters B, C, F, and H (see Fig E4). By contrast, no significant differences were observed in sputum IL-17 concentrations between healthy subjects and subjects of any cluster (data not shown).

**Fig E5:**
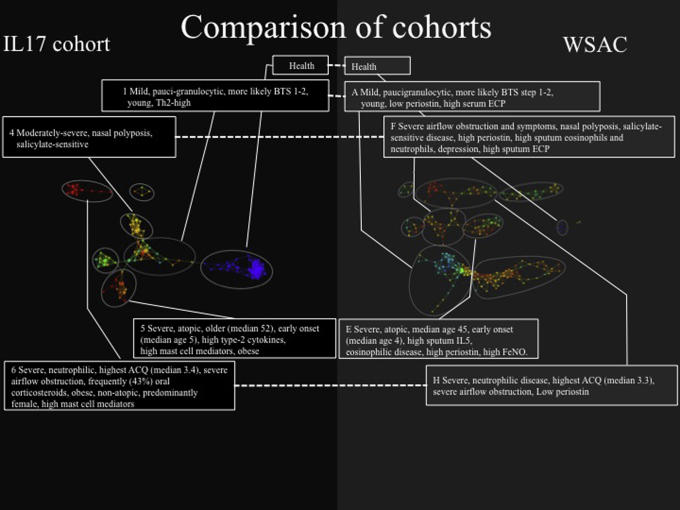


A qualitative comparison of these clusters and clusters we have previously identified by using similar methodology in a small and distinct cohort, the IL-17 cohort,^10^ is presented in Table E3 and Fig E5 in this article's Online Repository at www.jacionline.org. Clusters A, E, F, and H showed clear similarities to analogous clusters in the IL-17 cohort, although clusters B and C did not.

### Bayesian network analysis of combined data sets

**Fig 3:**
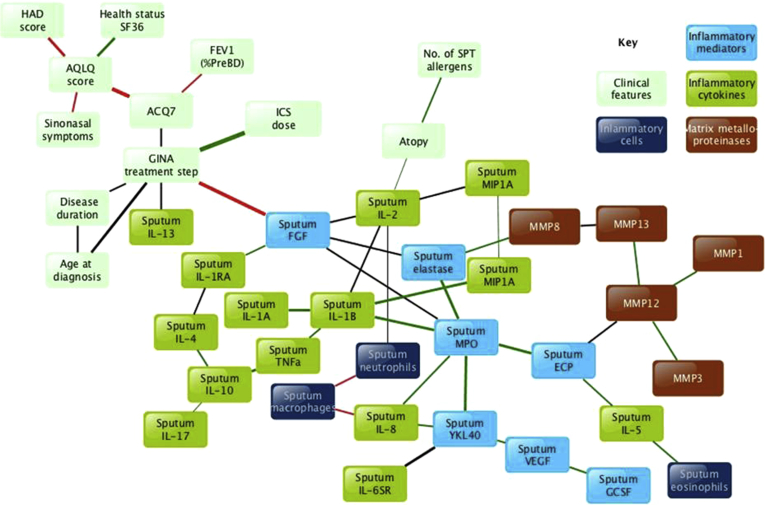
Bayesian belief network showing the strongest interactions between pathobiologic parameters across a range of clinical severities of asthma or health. Nodes without strong interactions are excluded. Line thickness represents the strength of the interaction (Euclidean distance). Line colors: *green*, positive associations; *red*, negative associations; *black*, nonlinear associations. *AQLQ*, Juniper Asthma Quality of Life Questionnaire; *BD*, bronchodilator; *FGF*, fibroblast growth factor; *GCSF*, Granulocyte-colony stimulating factor; *GINA*, Global Initiative for Asthma; *MPO*, myeloperoxidase; *SPT*, skin prick test; *VEGF*, vascular endothelial growth factor.

Next, to investigate the interactions between the diverse clinical, physiologic, and pathobiologic parameters in the data sets, we applied Bayesian network analysis to 74 nonredundant parameters in data from 173 participants from both cohorts with the highest quality sputum data (Fig 3). This Bayesian network provides a graphic representation of the probabilistic dependencies among the parameters and arises from the data by using machine learning inferred from the joint probability distributions of the data. In the figure the breadth of each line represents the strength of the interaction (Euclidean distance). Forty-one of the parameters were included in the model by the analysis, whereas 33 parameters without strong interactions were excluded from the model, including sex, Feno values, reversibility, peripheral blood counts, and serum periostin levels (see Table E2). Within the network, strong associations were observed between clinical parameters, and separately, strong associations were observed between pathobiologic parameters. However, a prominent feature of the network is a lack of associations between pathobiologic parameters and clinical parameters, with the exceptions of fibroblast growth factor, which is strongly negatively correlated with GINA treatment step, and atopic status, which is positively associated with sputum IL-2 levels.

**Fig E6:**
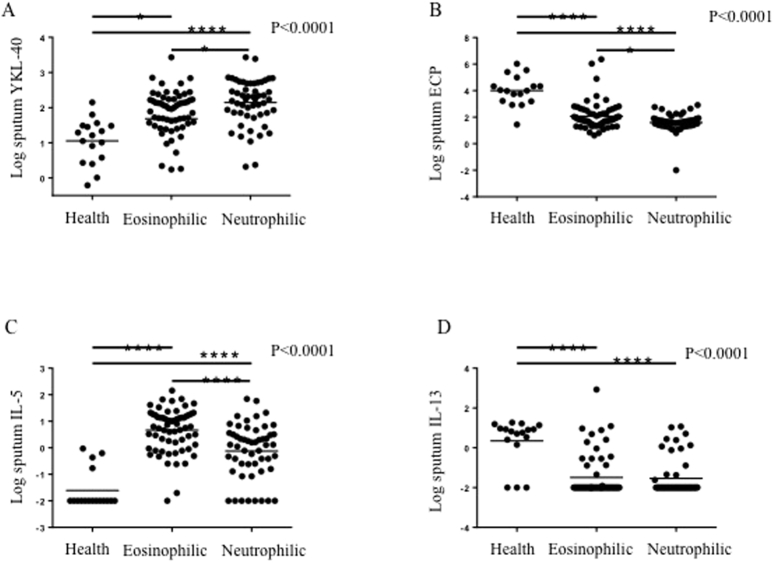


**Fig 4:**
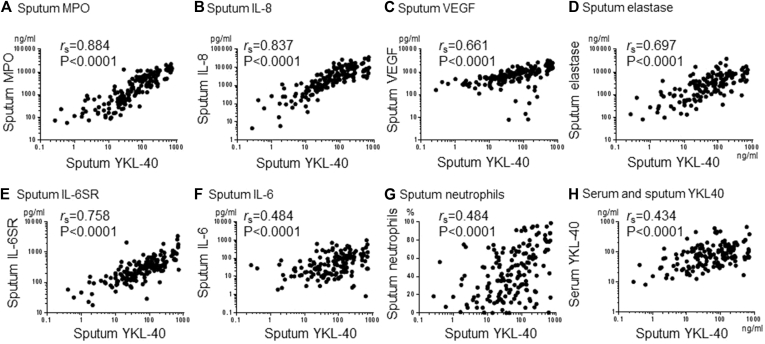
Inflammatory mediators associated with sputum YKL-40 levels. Spearman correlations between levels of sputum YKL-40 and sputum myeloperoxidase (*MPO*; **A**), IL-8 **(B)**, vascular endothelial growth factor (*VEGF*; **C**), elastase **(D)**, IL-6 soluble receptor (*IL-6SR*; **E**), IL-6 **(F)**, neutrophils **(G)**, and serum YKL-40 **(H)** are shown.

Sputum YKL-40 levels are highly connected within the network, particularly with levels of sputum MPO (Spearman *r*_s_ = 0.884, *P* < .0001), IL-8 (*r*_s_ = 0.837, *P* < .0001), and sputum IL-6 soluble receptor (*r*_s_ = 0.758, *P* < .0001; Fig 4, *A*, *B*, and *E*). Sputum YKL-40 levels correlated moderately with sputum neutrophil counts (*r*_s_ = 0.484, *P* < .0001; Fig 4, *G*) and were increased more in patients with neutrophilic versus eosinophilic disease (see Fig E6, *A*, in this article's Online Repository at www.jacionline.org). Sputum YKL-40 levels negatively correlated with lung function, particularly postbronchodilator FEV_1_ (*r*_s_ = −0.270, *P* = .0004, data not shown). Sputum and serum YKL-40 values are only moderately correlated (*r*_s_ = 0.434, *P* < .0001; Fig 4, *H*), which has implications for their utility as a serum biomarker.

**Fig E7:**
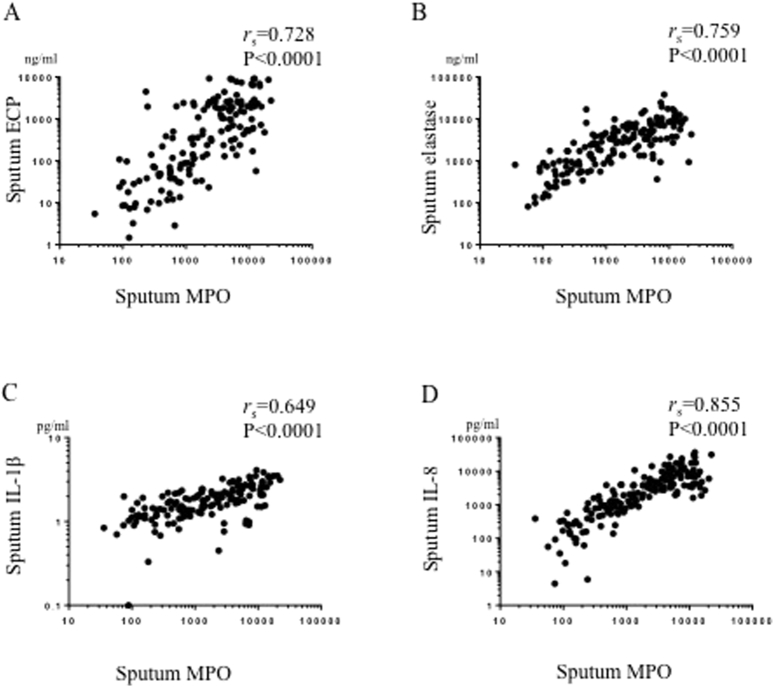


**Fig E8:**
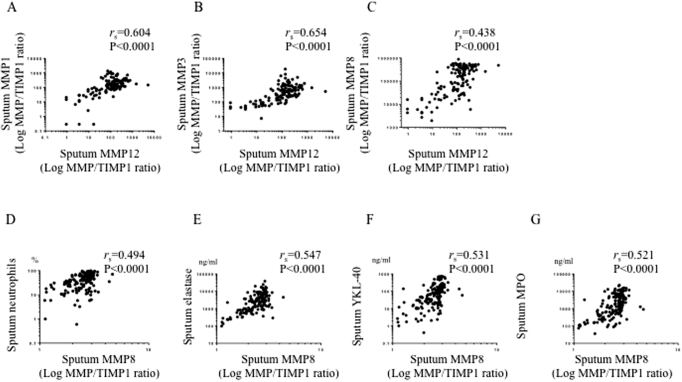


**Fig E9:**
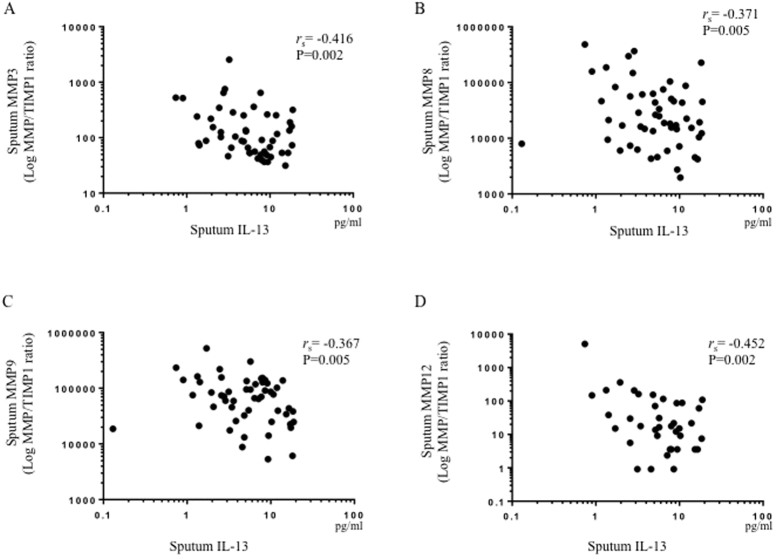


**Fig E10:**
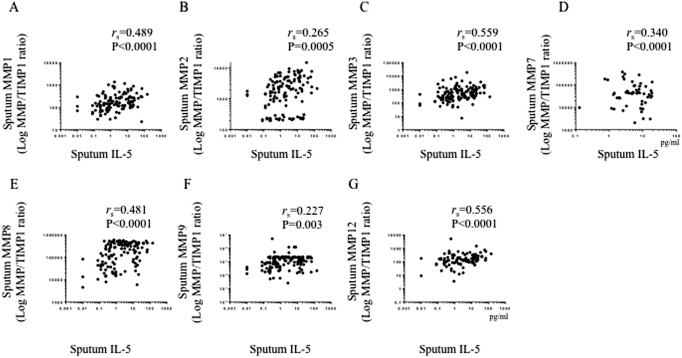


Levels of sputum MPO and sputum elastase, markers of neutrophilic airways inflammation, are highly connected within the network (Fig 3 and see Fig E7 in this article's Online Repository at www.jacionline.org). MMP12 levels are also highly connected, being positively associated with MMP1, MMP3, MMP8, and MMP13 levels (Fig 3 and see Fig E8 in this article's Online Repository at www.jacionline.org). Because MMP8 can induce the decoy receptor IL-13 receptor α2,^20^ a negative association between MMP concentrations and free IL-13 might be expected. Indeed, concentrations of these MMPs correlate negatively with sputum IL-13 levels (*r*_s_ = −0.371 to −0.452, see Fig E9 in this article's Online Repository at www.jacionline.org) but positively with sputum IL-5 levels (*r*_s_ = 0.481 to 0.559, *P* < .0001; see Fig E10 in this article's Online Repository at www.jacionline.org). MMP/tissue inhibitor of metalloproteinases 1 ratios are also associated with body mass index, particularly MMP1, MMP3, and MMP12 (*r*_s_ = 0.373, 0.303, and 0.311, respectively; *P* < .0001 each; data not shown).

**Fig E11:**
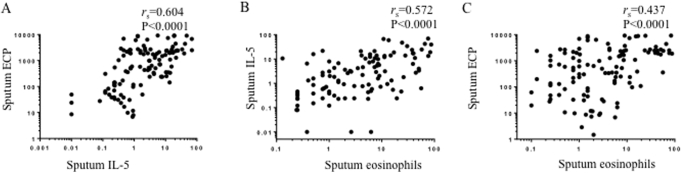


**Fig E12:**
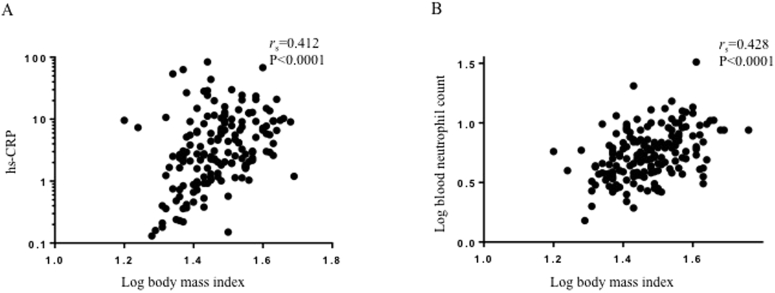


**Fig E13:**
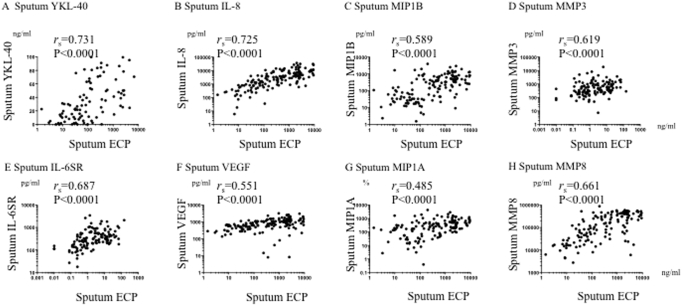


**Fig E14:**
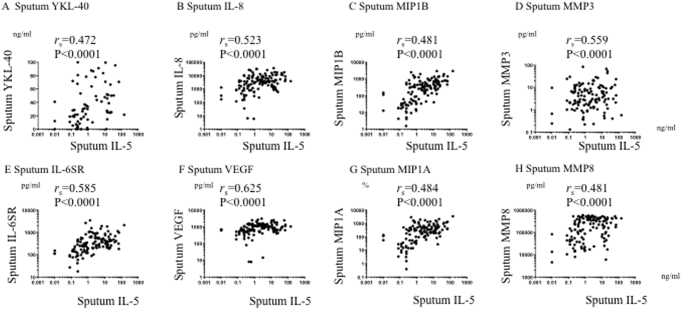


**Fig E15:**
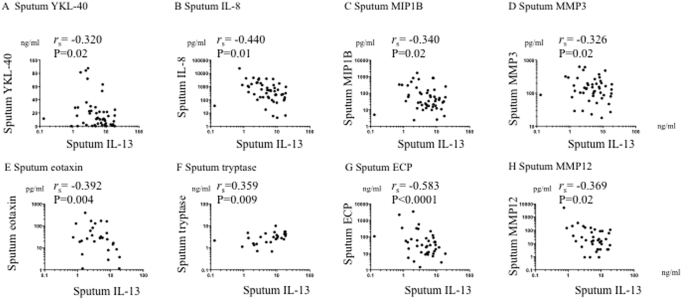


Although sputum IL-5 levels are strongly associated with sputum eosinophilia (*r*_s_ = 0.572, *P* < .0001) and sputum ECP levels (*r*_s_ = 0.604, *P* < .0001, see Figs E6 and E11 in this article's Online Repository at www.jacionline.org), sputum IL-13 levels are not highly correlated with sputum IL-5 levels (*P* = .6) and, conversely, are suppressed in both patients with neutrophilic and those with eosinophilic asthma (see Fig E6, *D*), suggesting that IL-13 production, which is strongly associated with GINA treatment step, is more steroid responsive than IL-5. These differences between associations of sputum ECP, IL-5, and IL-13 levels are further explored in Figs E11-E15 in this article's Online Repository at www.jacionline.org, which reveal that IL-5 is associated with a wider range of inflammatory markers than IL-13, including makers of neutrophilic inflammation (IL-8 and YKL-40). Thus disparities between IL-5 and IL-13 can have additional causes: given the association of IL-5 with ECP, which can be produced by both neutrophils and eosinophils,^21^ and that the eosinophil activation product major basic protein can increase eosinophil IL-8 production,^22^ the biology is complex, and IL-5 seems to have a broader effect than IL-13.

**Fig E16:**
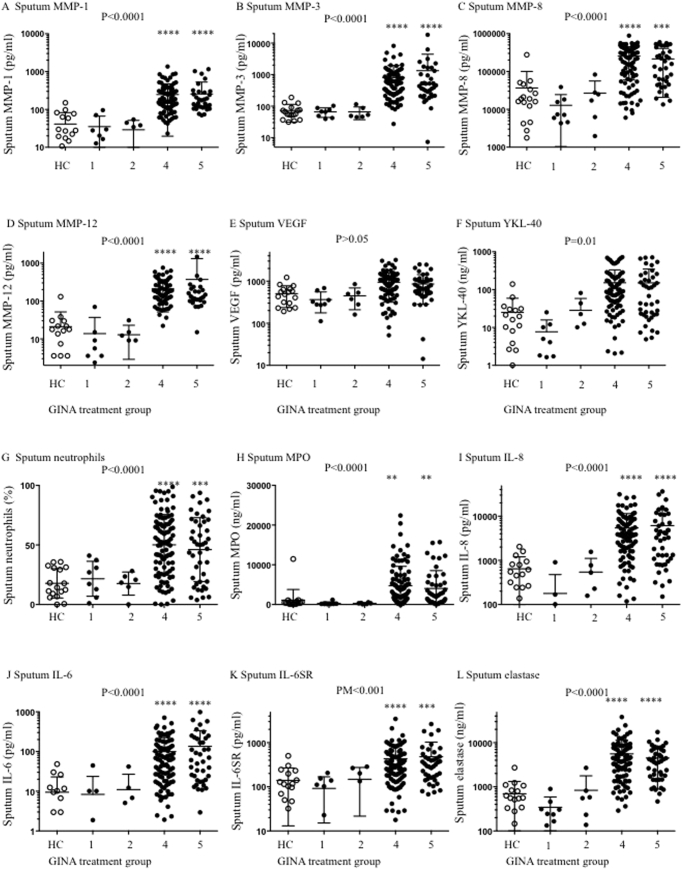


Fig E16 in this article's Online Repository at www.jacionline.org presents 12 key sputum parameters stratified according to GINA treatment group. For each of these parameters, no significant differences were observed between distributions for GINA step 4 compared with step 5 (receiving an additional median 10 mg/d oral prednisone), which would argue against our main observations being attributable wholly to therapeutic corticosteroids.

## Discussion

We have previously demonstrated the potential utility of topological and Bayesian analytic techniques to analyze high-dimensional flow cytometric data from a bronchoscopy study in a small asthma cohort in which we identified distinct multidimensional clinicopathologic clusters.^10^ Here we apply the same analytic approach to 2 much larger severe asthma cohorts, clustering patients by using only real-world assays already accessible to clinicians in tertiary referral centers for severe asthma. This provides endotypes that both relate to the underlying biology and are clinically meaningful.

In addition to the large study size and the statistically unbiased approach, a major strength of this study is the use of 2 geographically distinct cohorts. This provides true external validation of the derivation cohort model in contrast to studies that simply use a random split of a single data set, which provides only a weak and inefficient form of internal validation.^23^ The focus of our analyses is on differences within the spectrum of asthma. Although the number of healthy control subjects included is only modest, this has little effect on the identity of these endotypes, which are defined by comparison with all other subjects in the study. Although a larger sample size would enable a more detailed description of the clusters, the sensitivity of TDA to detect structure in small data sets and the external validation provide statistical confidence in the features described. A further strength of this study is the breadth of additional data on serum and airway inflammatory mediators also available for analysis, providing new insights into the roles of YKL-40 and MMPs in patients with severe asthma.

Several previous studies have investigated the existence of endotypes by using predominantly clinical parameters, which are straightforward to apply.^3^, ^7^, ^24^ Other studies have incorporated the additional dimension of sputum cell differential.^5^, ^6^, ^8^ Haldar et al^6^ observed 2 clusters characterized by discordance between symptoms and eosinophilic inflammation. Likewise, Wu et al^5^ included peripheral blood counts and bronchoalveolar lavage cell differentials when conducting a machine learning approach to analyze Severe Asthma Research Programme (SARP) data, identifying 6 asthma clusters. However, their analysis did not include induced sputum, and the composition of the clusters was again influenced mostly by clinical parameters because the selected markers of eosinophilic or neutrophilic inflammation provided only modest discriminating power in the model (INFOGAIN value, 0.12-0.16). Our analyses included sputum eosinophil and neutrophil counts; sputum subclass determination; measurement of Feno, periostin, high-sensitivity C-reactive protein, and serum IgE levels; and a restricted number of clinical parameters found to be nonredundant and have strong discriminatory power in our previous study.^10^ We identified 6 endotypes that could be replicated robustly in the validation cohort. The 2 dimensions of neutrophilic and eosinophilic inflammation are strongly influential in defining the shape of the data set and the composition of these clusters. As is clear from the Bayesian analysis, airway neutrophilia and eosinophilia represent 2 distinct inflammatory networks that likely contribute separately to asthma symptoms, again underlining the importance of considering these 2 specific dimensions when phenotyping an individual patient.

It is interesting that we identified more than 1 eosinophilic or neutrophilic cluster. Although airway eosinophilia and high periostin levels were common to clusters E and F, cluster F had the additional component of neutrophilic inflammation, leading to higher symptom scores and a mixed granulocytic subtype.^25^ Sputum ECP levels were significantly increased in group F (Table IV). ECP is a basic protein released during eosinophil degranulation and is highly correlated in our Bayesian analysis with both neutrophilic (sputum myeloperoxidase) and eosinophilic (sputum eosinophils and IL-5) inflammation (Fig 3 and see Fig E11). In the derivation cohort an additional neutrophilic cluster (ie, cluster G) was identified but not replicated, likely because of the small sample size, perhaps representing a milder spectrum of neutrophilic asthma with low periostin levels.

Similarities highlighted between clusters A, E, F, and H and analogous clusters we previously described in a separate cohort^10^ suggest our analytic approach produces consistent findings and that these clusters are likely related to fundamental differences in underlying disease mechanisms. The previous study aimed to investigate the full spectrum of clinical asthma. Thus 2 clusters of moderate disease in the IL-17 cohort were not replicated in the current study, which enrolled very few patients with moderate asthma, focusing instead on severe asthma, which is typical of our difficult asthma clinics.

How might our clusters relate to possible treatments? Our description of group E is consistent with descriptions by Haldar et al,^6^ Newby et al,^8^ Wu et al,^5^ Fingleton et al,^9^ and Hinks et al^10^ of a cluster of highly atopic, early-onset, eosinophilic asthma with high Feno values. We have shown this group to be characterized by high serum periostin and sputum IL-5 levels (see Fig E4 and Hinks et al^10^) despite high-dose corticosteroids, suggesting a likely response to agents, such as mepolizumab, targeting the IL-5 pathway. That the type 2 cytokines IL-5 and IL-13 are not associated in the Bayesian network and were not correlated in sputum suggests IL-13 is more susceptible to steroid therapy.^10^ Conversely, like others,^9^ we found mean serum IgE levels to be increased in all clusters, suggesting anti-IgE therapies might benefit several phenotypes. As before,^10^ we found no evidence of a significant dysregulation of airway IL-17 in any subgroup, implying this cytokine might not be a promising target.

In contrast to recent developments of therapies targeting T_H_2-mediated eosinophilic inflammation, there has been little progress in therapeutics for neutrophilic disease. Our Bayesian network highlights the prominent role of YKL-40 in neutrophilic inflammation. YKL-40 is a chitinase-like protein expressed by differentiated macrophages and is believed to regulate the magnitude of tissue injury and fibroproliferative repair in neutrophil granules.^26^ YKL-40 polymorphisms are associated with asthma, bronchial hyperresponsiveness, and reduced lung function.^27^, ^28^ Serum and sputum YKL-40 levels are increased in patients with severe asthma and correlate with disease severity, airway obstruction, and basement membrane thickness.^29^, ^30^ We observed a very strong correlation between sputum YKL-40 and sputum IL-8 levels, in addition to several other markers of neutrophilic inflammation, which is consistent with *ex vivo* observations that YKL-40 induces IL-8 from bronchial epithelia and stimulates smooth muscle proliferation.^31^ In addition, airway YKL-40 level increases in human allergen challenge and murine models implicate YKL-40 in airway eosinophilia and IL-5 production.^26^ In our data a moderate association (*r*_s_ = 0.477, *P* < .0001, data not shown) was seen with sputum IL-5 levels. Although genetic and *in vitro* data are suggestive, it remains to be determined whether YKL-40 plays a causative role in asthma or is simply a marker of extracellular tissue remodeling.

MMPs have also been implicated in tissue remodeling in asthmatic patients by human genetic studies,^32^, ^33^ murine emphysema models,^34^, ^35^, ^36^ and the findings of increased bronchoalveolar lavage MMP3 and MMP9 levels in patients with status asthmaticus^37^ and increased sputum MMP12 levels in asthmatic smokers.^38^ Our study, the largest to date, confirms and extends these findings with robust evidence of an increase in levels of specific MMPs in patients with severe asthma. These include MMP1, MMP3, and MMP8, the 3 secreted type 3 collagenases able to degrade collagen at neutral pH. We show that these are strongly associated with neutrophilic inflammation, dysregulated in obese asthmatic patients, and correlated positively with sputum IL-5 levels but negatively with sputum IL-13 levels, perhaps because MMP8 cleaves the IL-13 decoy receptor (IL-13 receptor α2).^20^

In conclusion, we have identified and replicated 6 clinicopathobiologic clusters using assays and sputum induction available in clinical practice. Our data underline a disconnect between clinical features and underlying inflammation, suggest IL-5 production is relatively steroid insensitive, and highlight the roles of YKL-40 in neutrophilic inflammation and specific MMPs in severe asthma.

**Key messages**

• We have identified and replicated 6 clinicopathobiologic asthma endotypes.
• MMP1, MMP3, MMP8, and MMP12 levels are increased in patients with severe asthma and associated with sputum IL-5 levels.
• YKL-40 is strongly implicated in neutrophilic asthma.

## Figures and Tables

**Table I:** Demographics of clusters in the derivation cohort

Parameters	Healthy control subjects	Cluster							
A	B	C	D	E	F	G	H
No.∗	8	30	7	13	4	13	17	37	19
Demographics									
Sex (male/female), no. (%)	3 (38)/5 (72)	9 (30)/21 (70)	4 (57)/3 (43)	3 (23)/10 (77)	2 (50)/2 (50)	6 (46)/7 (54)	9 (53)/8 (47)	13 (35)/24 (65)	9 (47)/10 (53)
Age (y), median (range)	33.5 (21-53)	38 (22-65)	60 (39-67)	44 (21-57)	54.5 (23-61)	45 (26-62)	57 (29-68)	51 (23-69)	58 (43-71)
Pulmonary function									
FEV_1_ (% predicted, pre-BD)†	89 (84-98)	91 (83-105)	58 (53-64)	80 (72-94)	99 (63-115)	64 (58-83)	40 (31-64)	74 (55-84)	44 (35-59)
FEV_1_ reversibility (%)	0 (0.0-1.8)	6.8 (4.1-9.3)	16 (5.7-23)	5.8 (2.6-11)	4.7 (0.68-11)	8.7 (2.7-20)	12 (8.2-24)	11 (3.3-20)	14 (3.3-25)
FEV_1_ (% predicted, post-BD)	92 (84-98)	96 (87-112)	67 (63-76)	85 (70-104)	112 (71-122)	75 (64-87)	52 (30-77)	82 (66-88)	54 (44-66)
Exhaled nitric oxide (ppb, at 50 L/s)	13 (11-17)	26 (12-52)	25 (19-45)	11 (9.5-19)	20 (16-35)	33 (11-73)	22 (17-46)	19 (10-29)	17 (10-30)
Clinical									
Atopy (positive skin test response, yes/no), no. (%)	4 (50)/4 (50)	22 (73)/8 (27)	4 (57)/3 (43)	5 (38)/8 (62)	3 (75)/1 (25)	11 (82)/2 (18)	12 (71)/5 (29)	22 (59)/15 (41)	13 (68)/6 (32)
No. of allergens eliciting positive skin test responses	1 (0-23)	3 (0-5)	3 (0-4)	0 (0-4)	2 (1-4)	2 (1-5)	3 (0-6)	2 (0-4)	2 (0-3)
Peripheral eosinophil count (10^9^/L)	0.1 (0.1-0.3)	0.2 (0.1-0.4)	0.2 (0.1-0.4)	0.3 (0.1-0.6)	0.2 (0.1-1.3)	0.6 (0.4-0.7)	0.4 (0.1-0.7)	0.2 (0.1-0.2)	0.2 (0.1-0.3)
Total IgE (IU/mL)	230 (79-280)	120 (30-260)	88 (23-210)	34 (11-110)	130 (37-190)	68 (13-812)	380 (110-1400)	92 (12-260)	130 (28-290)
Body mass index (kg/m^2^)	23.5 (22.4-25.6)	31.3 (26.7-35.7)	28.0 (27.6-35.5)	36.4 (32.4-41.7)	34.6 (25.6-37.9)	25.9 (23.3-29.0)	28 (25.8-37.2)	25.5 (24.3-29.8)	30.9 (28.6-36.5)
Smoking status									
Never, no. (%)	5 (72)	15 (50)	5 (71)	4 (31)	4 (100)	7 (54)	10 (59)	17 (46)	10 (53)
Former, no. (% [mean pack years])	3 (38 [3.3])	12 (40 [14])	2 (29 [29])	7 (54 [16])	0 (0)	6 (46 [3])	6 (35 [22])	14 (38 [16])	5 (26 [13])
Current, no. (% [mean pack years])	0 (0)	3 (10 [13])	0 (0)	2 (15 [23])	0 (0)	0 (0)	1 (5.9 [6.5])	6 (16 [28])	4 (21 [35])
Duration of asthma (y)	NA	19 (5-31)	30 (15-49)	21 (6-32)	12 (9.3-20)	29 (21-42)	34 (25-47)	29 (18-44)	43 (23-47)
ACQ7 score	NA	1.6 (0.9-2.7)	2.3 (1.7-4.1)	2.7 (2.1-3.7)	2.1 (0.43-2.7)	2.9 (1.7-4.0)	3.1 (2.3-3.9)	3.3 (2.4-3.9)	3.3 (2.5-4.2)
Treatment									
Inhaled steroid dose (equivalent μg of BDP)	0	1240 (0-2160)	2400 (1600-2400)	1440 (1220-2080)	1640 (400-1860)	1600 (800-1840)	2000 (1760-2000)	1640 (1280-2080)	1600 (920-2300)
Maintenance oral corticosteroids (yes/no), no. (%)	0 (0)/8 (0)	5 (17)/25 (83)	0 (0)/7 (100)	5 (38)/8 (62)	2 (50)/2 (50)	6 (46)/7 (54)	5 (29)/12 (71)	12 (32)/25 (68)	3 (16)/16 (84)
Inflammatory subtype, no. (%)									
Neutrophilic	0 (0)	0 (0)	3 (43)	3 (23)	0 (0)	0 (0)	6 (35)	8 (22)	13 (68)
Eosinophilic	1 (13)	6 (20)	3 (43)	3 (23)	2 (50)	0 (0)	4 (24)	10 (27)	1 (5)
Mixed granulocytic	0 (0)	0 (0)	1 (14)	1 (8)	0 (0)	9 (69)	4 (24)	3 (8)	2 (10)
Paucigranulocytic	7 (87)	24 (80)	0 (0)	6 (46)	2 (50)	4 (31)	3 (18)	16 (43)	3 (16)
Sputum cell differential (%)									
Macrophages	70 (58-85)	70 (57-76)	17 (7.7-28)	44 (28-59)	64 (26-68)	41 (25-51)	13 (5.3-48)	36 (26-55)	25 (9.7-29)
Neutrophils	12 (7.7-30)	19 (13-28)	66 (57-84)	52 (40-61)	21 (22-27)	30 (22-41)	65 (47-91)	53 (34-64)	71 (64-88)
Eosinophils	0.75 (0.60-1.3)	1.3 (0.0-2.6)	5.3 (0.3-13)	0.25 (0.0-1.3)	2 (0.13-17)	14 (1.8-43)	2.8 (1.2-8.1)	1 (0.3-6.0)	0.75 (0.06-1.38)
Lymphocytes	0.0 (0.0-0.0)	0.0 (0.0-0.38)	0.0 (0.0-0.0)	0.0 (0.0-0.25)	0.0 (0.0-0.06)	0.0 (0.0-0.25)	0 (0.0-0.31)	0.15 (0.0-0.30)	0.0 (0.0-0.19)
Epithelial	2.4 (1.5-11)	8.0 (2.5-12)	1.3 (0.9-7.0)	4.0 (1.0-6.0)	9.1 (0.38-14)	3.4 (2.2-4.9)	1.3 (0.25-2.4)	2.8 (1.5-9.8)	1.2 (0.31-3.7)

The inflammatory subtype is based on sputum differentials by using the following cut points: neutrophilic, greater than 61%; eosinophilic, greater than 3%. Percentages shown are derived from those subjects with valid data.

*ACQ*, Asthma Control Questionnaire^11^; *BD*, bronchodilator; *BDP*, beclomethasone dipropionate; *CT*, computed tomography; *FVC*, forced vital capacity; *GINA*, Global Initiative for Asthma; *NA*, not available; *PEFR*, peak expiratory flow rate.

∗ Because some subjects were outliers, not all are assigned to clusters A through H.

† Values are medians with interquartile ranges, unless stated otherwise.

**Table II:** Demographics of clusters in the validation cohort

Parameters	Healthy control subjects	Cluster						
a	b	c	e	f	h	i
No.∗	13	4	9	7	5	19	9	5
Demographics								
Sex (male/female), no. (%)	5 (8)/8 (62)	3 (75)/1 (25)	7 (78)/2 (22)	3 (43)/4 (57)	1 (20)/4 (80)	12 (63)/7 (37)	2 (22)/7 (72)	3 (60)/2 (40)
Age (y), median (range)	34 (18-53)	34 (23-51)	61 (29-79)	44 (30-62)	61 (45-71)	51 (29-79)	57 (30-73)	45 (41-50)
Pulmonary function								
FEV_1_ (% predicted, pre-BD)†	104 (96-108)	103 (95-109)	57 (52-62)	73 (68-78)	50 (49-52)	60 (51-78)	48 (44-69)	75 (75-85)
FEV_1_ reversibility (%)	0 (0.0-0.0)	2.1 (−0.3-4.7)	8.4 (1.2-17)	11 (5.3-13)	5.8 (2.6-17)	13.3 (5.7-15)	7.5 (6.2-14)	9.3 (5.0-9.4)
FEV_1_ (% predicted, post BD)	104 (96-108)	105 (97-111)	61 (53-73)	78 (73-86)	53 (51-62)	70 (58-83)	53 (50-73)	81 (80-87)
Exhaled nitric oxide (ppb, at 50 L/s)	14 (11-18)	39 (28-72)	32 (17-68)	26 (17-53)	72 (17-98)	32 (18-64)	27 (16-52)	28 (14-51)
Clinical								
Atopy (positive skin test response, yes/no), no. (%)	4 (31)/9 (69)	3 (75)/1 (25)	8 (89)/1 (11)	6 (83)/1 (17)	4 (80)/1 (20)	13 (68)/6 (32)	5 (56)/44 (44)	4 (80)/1 (20)
No. of allergen eliciting positive skin test responses	0 (0-0)	3.5 (2-6)	3 (1-4)	2 (2-4)	2 (1-3)	3 (0-4.5)	1 (0-4)	5 (5-5)
Peripheral eosinophil count (10^9^/L)	0.1 (0.1-0.1)	0.1 (0.1-0.2)	0.5 (0.3-0.5)	0.3 (0.1-0.4)	0.2 (0.1-0.3)	0.5 (0.2-0.9)	0.3 (0.0-0.4)	0.5 (0.5-0.6)
Total IgE (IU/mL)	21 (8.8-52)	77 (34-130)	116 (69-136)	145 (79-1500)	149 (100-860)	130 (54-170)	73 (32-540)	266 (140-400)
Body mass index (kg/m^2^)	24.3 (21.9-28.4)	29.4 (26.1-31.5)	26.4 (25.9-29.0)	30.9 (27.8-33.5)	27.3 (26.4-28.4)	29.1 (26.4-32.1)	26.2 (41.0-29.1)	32.1 (27.2-34.2)
Smoking status								
Never, no. (%)	11 (85)	1 (25)	2 (22)	4 (57)	2 (40)	10 (53)	6 (67)	2 (60)
Former, no. (% [mean pack years])	2 (15 [2.5])	3 (75 [6])	7 (78 [25])	3 (43 [19])	2 (40% [25])	8 (42 [20])	3 (33 [17])	3 (40% [16])
Current, no. (% [mean pack years])	0 (0)	0 (0)	0 (0)	0 (0)	1 (20 [32])	1 (5.3 [32])	0 (0)	0 (0)
Duration of asthma (y)	NA	16 (12-22)	26 (14-38)	30 (19-47)	13 (5-16)	33 (18-46)	22 (6-41)	41 (21-41)
ACQ7 score	NA	0.76 (0.43-1.2)	3.4 (2.9-4.0)	2.3 (1.9-3.2)	3.6 (3.0-4.1)	2.9 (2.4-3.6)	3.4 (2.7-4.0)	3.1 (3.0-3.3)
Treatment								
Inhaled steroid dose (equivalent μg of BDP)	0	3280 (2280-3940)	1600 (1600-2000)	2880 (1840-4440)	2000 (2000-2880)	1600 (1600-2000)	2240 (1270-2850)	2000 (2000-2000)
Maintenance oral corticosteroids (yes/no), no. (%)	0 (0)/13 (100)	0 (0)/4 (100)	4 (44)/5 (56)	2 (29)/5 (71)	2 (40)/3 (60)	6 (32)/13 (68)	3 (33)/6 (66)	3 (60)/2 (40)
Inflammatory subtype, no. (%)								
Neutrophilic	0 (0)	0 (0)	1 (11)	2 (29)	0 (0)	6 (32)	3 (33)	0 (0)
Eosinophilic	0 (0)	0 (0)	5 (56)	2 (29)	3 (60)	9 (47)	2 (22)	2 (40)
Mixed granulocytic	0 (0)	0 (0)	1 (11)	0 (0)	0 (0)	1 (5)	3 (33)	0 (0)
Paucigranulocytic	13 (100)	4 (100)	2 (22)	3 (43)	2 (40)	3 (16)	1 (11)	3 (60)
Sputum cell differential (%)								
Macrophages	82 (69-89)	70 (63-78)	26 (19-54)	34 (25-39)	52 (12-67)	30 (22-51)	24 (17-31)	84 (75-89)
Neutrophils	18 (11-33)	26 (17-34)	50 (42-50)	45 (39-53)	18 (9.9-23)	50 (32-70)	67 (51-76)	16 (3.8-17)
Eosinophils	0.0 (0.0-0.2)	0.0 (0.0-0.13)	11 (3.8-25)	0.69 (0.38-30)	42 (5.4-79)	4.9 (2.0-13)	7.4 (1.8-19)	3.3 (0.29-6.7)
Lymphocytes	0.2 (0.0-0.63)	0.1 (0.05-0.68)	0.94 (0.5-1.2)	0.69 (0.25-1.5)	0.25 (0.0-0.53)	0.63 (0.5-1.3)	1.4 (1.0-1.6)	0.38 (0.19-0.50)
Epithelial	0.1 (0.0-0.3)	1.0 (1.0-1.5)	0.5 (0.22-1.0)	0.69 (0.16-0.94)	0.69 (0.0-2.1)	1.8 (0.38-2.0)	0.5 (0.25-0.76)	0.19 (0.10-1.6)

The inflammatory subtype is based on sputum differentials by using the following cut points: neutrophilic, greater than 61%; eosinophilic, greater than 3%. Percentages given are derived from those subjects with valid data.

*ACQ*, Asthma Control Questionnaire^11^; *BD*, bronchodilator; *BDP*, beclomethasone dipropionate; *CT*, computed tomography; *FVC*, forced vital capacity; *GINA*, Global Initiative for Asthma; *NA*, not available; *PEFR*, peak expiratory flow rate.

∗ Because some subjects were outliers, not all are assigned to clusters a through i.

† Values are medians with interquartile ranges, unless stated otherwise.

**Table III:** Clinical and pathologic features found to be associated with patients with severe asthma compared with healthy subjects

Derivation data set					Validation data set			
Feature	Healthy subjects	Patients with severe asthma	K-S tests		Healthy subjects	Patients with severe asthma	K-S tests	
K-S score	*P* value	K-S score	*P* value
No.∗	8	121			13	50		
Increased in asthmatic patients compared with healthy subjects								
Reversibility (%)	0.0 (0.0-1.8)	10 (2.9-21)	0.590	.01	0.0 (0.0-0.0)	8.8 (3.3-14)	0.841	<.001
BMI†	23.5 (22.4-25.6)	31.2 (26.7-37.3)	0.566	.02	24.3 (21.9-28.4)	29.0 (26.0-32.2)	0.452	.03
HAD score	4.0 (1.8-7.8)	15 (10-22)	0.597	.01	3.0 (0.0-5.0)	16 (9.0-25)	0.637	<.001
HAD Depression score	1 (1-3)	9 (6-11)	0.680	.01	2 (0-2)	8.5 (5.3-12)	0.734	<.001
Nasal dysfunction‡	0.35 (0.09-0.39)	0.48 (0.42-0.72)	0.558	.02	0.0 (0.0-0.43)	0.39 (0.13-0.53)	0.708	<.001
SNOT-20	12 (1.5-27)	35 (24-48)	0.507	.04	0.0 (0.0-3.0)	37 (20-53)	0.739	<.001
Serum YKL-40 (ng/mL)†	17 (13-22)	83 (55-140)	0.787	<.001	27 (19-37)	110 (67-160)	0.739	<.001
Blood neutrophil count (10^9^/L)†	3.9 (3.2-4.5)	6.1 (4.3-8.4)	0.603	.008	2.9 (2.7-4.0)	5.6 (4.4-8.5)	0.545	.004
Sputum neutrophil count (%)	12 (7.7-30)	51 (28-68)	0.571	.01	18 (11-33)	45 (29-67)	0.732	<.001
Sputum MMP3§	3.7 × 10^−4^ (3.7 × 10^−4^-3.9 × 10^−4^)	2.6 × 10^−2^ (1.3 × 10^−2^-5.9 × 10^−2^)	0.931	<.001	1.6 × 10^−4^ (9.9 × 10^−5^-2.3 × 10^−4^)	3.2 × 10^−2^ (1.5 × 10^−2^-5.9 × 10^−2^)	0.902	<.001
Sputum MMP12§	7.0 × 10^−5^ (6.3 × 10^−6^-1.4 × 10^−4^)	1.0 × 10^−2^ (5.9 × 10^−3^-1.6 × 10^−2^)	0.774	.002	3.7 × 10^−5^ (1.2 × 10^−5^-5.5 × 10^−5^)	1.0 × 10^−2^ (5.1 × 10^−3^-1.9 × 10^−2^)	0.878	<.001
Sputum MMP8§	0.12 (0.049-0.21)	9.6 (2.2-27)	0.628	.02	0.04 (0.030-0.077)	21 (5.4-28)	0.854	<.001
Sputum MMP1§	1.6 × 10^−4^ (3.3 × 10^−5^-3.6 × 10^−4^)	1.1 × 10^−2^ (6.2 × 10^−3^-2.1 × 10^−2^)	0.627	.02	5.7 × 10^−5^ (3.6 × 10^−5^-1.5 × 10^−4^)	1.2 × 10^−2^ (6.7 × 10^−3^-2.0 × 10^−2^)	0.732	<.001
Sputum VEGF†	230 (220-280)	700 (470-1100)	0.814	.001	580 (520-710)	1000 (620-1300)	0.519	.02
Sputum IL-6 soluble receptor (pg/mL)†	41 (8.0-99)	260 (140-430)	0.607	.03	130 (100-215)	480 (260-840)	0.623	.002
Sputum IL-6 (pg/mL)†	0.0 (0.0-0.0)	50 (17-120)	0.873	<.001	10 (2.3-19)	55 (15-170)	0.567	.007
Sputum IL-5 (pg/mL)†	0.0 (0.0-0.0)	0.76 (0.18-6.4)	0.627	.02	0.0 (0.0-0.080)	3.1 (0.72-9.8)	0.714	<.001
Sputum IL-8 (pg/mL)†	190 (100-420)	3300 (1000-8200)	0.676	.01	620 (380-880)	4000 (2100-7100)	0.756	<.001
Sputum YKL-40 (ng/mL)†	3.2 (2.5-8.7)	65 (20-150)	0.647	.02	21 (14-31)	150 (48-270)	0.738	<.001
Decreased in asthmatic patients compared with healthy subjects								
FEV_1_/FVC ratio	80 (77-83)	66 (54-72)	0.752	<.001	85 (84-88)	66 (58-74)	0.785	<.001
FEV_1_ (% predicted, pre-BD)	89 (84-98)	68 (49-84)	0.560	.008	100 (96-108)	60 (49-75)	0.918	<.001
FEV_1_ (% predicted, post-BD)	92 (84-98)	78 (58-90)	0.608	.02	100 (96-110)	68 (52-81)	0.857	<.001
AQLQ score	7 (7-7)	3.7 (3.0-4.8)	0.936	<.001	7.0 (7.0-7.0)	4.1 (3.1-4.9)	0.959	<.001
SF-36	89 (71-91)	42 (27-61)	0.776	<.001	89 (85-92)	46 (33-65)	0.841	<.001
Sputum macrophage count (%)	70 (58-85)	36 (23-56)	0.646	.001	82 (69-89)	30 (19-52)	0.837	<.001
Sputum TIMP-1 (ng/mL)†	1.7 × 10^5^ (7.2 × 10^4^-2.5 × 10^6^)	1.2 × 10^4^ (5.2 × 10^3^-3.8 × 10^4^)	0.725	.005	4.7 × 10^5^ (2.3 × 10^5^-1.5 × 10^6^)	1.7 × 10^4^ (6.3 × 10^3^-3.8 × 10^4^)	0.860	<.001
Sputum IL-2 (pg/mL)†	1.8 (0.27-2.7)	0.0 (0.0-0.0)	0.627	.02	0.95 (0.0-3.1)	0.0 (0.0-0.0)	0.612	.003
Sputum IL-1RA (pg/mL)†	2.6 × 10^4^ (2.4 × 10^4^-2.8 × 10^4^)	2.7 × 10^3^ (0.0-1.2 × 10^4^)	0.941	<.001	2.6 × 10^4^ (2.2 × 10^4^-3.5 × 10^4^)	1.4 × 10^4^ (1.1 × 10^4^-1.8 × 10^4^)	0.745	<.001
Sputum FGF (pg/mL)	53 (43-62)	0.0 (0.0-0.60)	0.941	<.001	48 (44-57)	1.2 (2.5 × 10^−3^-1.2)	0.860	<.001

These features were found to differ significantly between healthy subjects and patients with severe asthma (British Thoracic Society steps 4 and 5) in both training and validation data sets.

*AQLQ*, Juniper Asthma Quality of Life Questionnaire; *BD*, bronchodilator; *BDP*, beclomethasone dipropionate equivalent; *BMI*, body mass index; *FGF*, fibroblast growth factor; *FVC*, forced vital capacity; *GINA*, Global Initiative for Asthma; IL-1RA, IL-1 receptor antagonist; *MPO*, myeloperoxidase; SF-36, Short-Form 36 Health Survey; *SNOT-20*, Sino-Nasal Outcome Test 20; *TIMP-1*, tissue inhibitor of metalloproteinases 1; *VEGF*, vascular endothelial growth factor.

∗ Values are medians with interquartile ranges, unless stated otherwise.

† Statistical tests were performed on transformed data.

‡ “Nasal dysfunction” is a composite average score on a scale of 0 to 1 derived from SNOT-20 scores, hyposmia, and rhinosinusitis.

§ MMPs are expressed as a ratio to tissue inhibitor of metalloproteinases values; statistical tests were performed on transformed data.

**Table IV:** Definitions of clusters in the derivation and validation data sets

Derivation data set					Validation data set				
Cluster	Features of cluster	K-S tests		Comments	Cluster	Features of cluster	K-S tests		Comments
K-S score	*P* value	K-S score	*P* value
A	Young, mild, paucigranulocytic				a				
	Lower serum periostin level	0.365	.003	More likely GINA step 2 (*P* < .001)		Lower serum periostin level	0.758	.03	More likely GINA step 2 (*P* = .001)
				Predominantly paucigranulocytic sputum (*P* < .001)					Predominantly paucigranulocytic sputum (*P* = .04)
									
				Lowest median ACQ7 score (1.6)					Lowest median ACQ7 score (0.8)
				Youngest median age (38 y)					Youngest median age (34 y)
B	Older, sinonasal disease				b				
	Higher serum periostin level	0.746	.001	Oldest median age (60 y)		Higher serum periostin level	0.709	<.001	Joint oldest median age (61 y)
	Higher sputum MMP3 level	0.610	.03	Highest median HAD score (18)		Higher sputum MMP3 level	0.562	.04	Highest median HAD score (27)
	Higher SNOT-20 score	0.533	.04			Higher SNOT-20 score	0.803	<.001	
C	Obese, high MMP level				c				
	Higher sputum MMP1 level	0.498	.02	Highest BMI (36.4)		Higher sputum MMP levels	0.735	.006	Highest BMI (30.9) after group i
	Higher sputum MMP8 level	0.481	.03						
	Higher sputum MMP2 level	0.474	.03						
	Lower serum periostin level	0.802	<.001			Lower serum periostin level	0.746	.002	
D	This group was not replicated in the validation set								
	Higher serum periostin level	0.780	.02						
	Higher HAD Depression score	0.695	.04						
E	Steroid resistant T_H_2 mediated, eosinophilic				e				
	Higher serum periostin level	0.811	<.001	More likely eosinophilic sputum class (*P* = .03)		Higher serum periostin level	0.862	.002	Predominantly (60%) eosinophilic sputum class
	Higher eosinophilia	0.524	.008			Higher eosinophilia	0.981	.05	
	Higher sputum IL-5 level	0.503	.01	Highest median Feno value (33 ppb)		Higher sputum IL-5 level	1.00	.04	Highest median Feno value (72 ppb)
				Youngest median age of onset (4 y)					Youngest median age of onset (13 y)
F	Mixed granulocytic inflammation with severe obstruction				f				
	Higher serum periostin level	0.781	<.001			Higher serum periostin level	0.569	<.001	
	Higher sputum ECP level	0.503	.005			Higher sputum ECP level	0.413	.04	
	Higher sputum neutrophil count	0.465	.003			Higher sputum neutrophil count	0.373	.04	
	Higher sputum eosinophil count	0.391	.0197			Higher sputum eosinophil count	0.438	.01	
	Higher HAD Depression score	0.367	.03			Higher HAD Depression score	0.379	.04	
	Lower FEV_1_ (% predicted, pre-BD)	0.495	.001			Lower FEV_1_ (% predicted, pre-BD)	0.379	.04	
	Lower FEV_1_/FVC ratio	0.479	.002			Lower FEV_1_/FVC ratio	0.463	.005	
	Lower sputum macrophage counts	0.409	.01			Lower sputum macrophage counts	0.438	.01	
G	This group was not replicated in the validation set								
	Higher neutrophilia	0.304	.05						
	Higher sputum osteopontin level	0.300	.05						
	Higher blood neutrophil count	0.292	.02						
	Higher ACQ7 score	0.285	.02						
	Lower serum periostin level	0.426	<.001						
	Lower sputum MMP9 level	0.378	.006						
	Lower sputum α2M level	0.350	.01						
	Lower sputum FGF level	0.303	.05						
	Lower SF-36 score	0.344	.003						
	Lower AQLQ score	0.289	.02						
H	Neutrophilic disease with severe obstruction, low periostin level								
	Higher sputum neutrophil count	0.667	<.001	More likely neutrophilic sputum class (*P* < .0001)		Higher sputum neutrophil count	0.639	.003	More likely neutrophilic sputum class (*P* = .005)
	Lower FEV_1_ (% predicted, pre-BD)	0.569	<.001			Lower FEV_1_ (% predicted, pre-BD)	0.490	.04	
	Lower FEV_1_ (% predicted, post-BD)	0.562	<.001	Very low FEV_1_ (44% of predicted value, pre-BD)		Lower FEV_1_ (% predicted, post-BD)	0.519	.03	Lowest FEV_1_ (48% of predicted value, pre-BD)
	Lower periostin level	0.495	<.001	Highest median ACQ score (3.3)		Lower periostin level	0.639	.003	High median ACQ score (3.4)
					i	This group was not replicated in the validation set			
						Higher SNOT-20 score	0.677	.03	
						Higher sputum macrophage counts	0.631	.05	
						Lower AQLQ emotional score	0.677	.03	
						Lower periostin level	0.646	.04	

Features of clusters were identified in the training and validation sets by using TDA. Features are listed that differ significantly in the cluster when compared with all other subjects in the same cohort.

*α2M*, α2-Macroglobulin; *AQLQ*, Juniper Asthma Quality of Life Questionnaire; *BD*, bronchodilator; *BDP*, beclomethasone dipropionate equivalent; *BMI*, body mass index; *FGF*, fibroblast growth factor; *FVC*, forced vital capacity; *GINA*, Global Initiative for Asthma; *MPO*, myeloperoxidase; *SNOT-20*, Sino-Nasal Outcome Test 20.

## Supplementary data

Online Repository Data

Tables E1-E3

## References

[1] Global Initiative for Asthma (GINA) Global strategy for asthma management and prevention 2015. Available at: http://www.ginasthma.org/. Accessed January 8, 2016.

[2] Anderson G.P. (2008). Endotyping asthma: new insights into key pathogenic mechanisms in a complex, heterogeneous disease. Lancet.

[3] Lotvall J., Akdis C.A., Bacharier L.B., Bjermer L., Casale T.B., Custovic A. (2011). Asthma endotypes: a new approach to classification of disease entities within the asthma syndrome. J Allergy Clin Immunol.

[4] Moore W.C., Bleecker E.R., Curran-Everett D., Erzurum S.C., Ameredes B.T., Bacharier L. (2007). Characterization of the severe asthma phenotype by the National Heart, Lung, and Blood Institute's Severe Asthma Research Program. J Allergy Clin Immunol.

[5] Wu W., Bleecker E., Moore W., Busse W.W., Castro M., Chung K.F. (2014). Unsupervised phenotyping of Severe Asthma Research Program participants using expanded lung data. J Allergy Clin Immunol.

[6] Haldar P., Pavord I.D., Shaw D.E., Berry M.A., Thomas M., Brightling C.E. (2008). Cluster analysis and clinical asthma phenotypes. Am J Respir Crit Care Med.

[7] Boudier A., Curjuric I., Basagana X., Hazgui H., Anto J.M., Bousquet J. (2013). Ten-year follow-up of cluster-based asthma phenotypes in adults. A pooled analysis of three cohorts. Am J Respir Crit Care Med.

[8] Newby C., Heaney L.G., Menzies-Gow A., Niven R.M., Mansur A., Bucknall C. (2014). Statistical cluster analysis of the British Thoracic Society Severe refractory Asthma Registry: clinical outcomes and phenotype stability. PLoS One.

[9] Fingleton J., Travers J., Williams M., Charles T., Bowles D., Strik R. (2015). Treatment responsiveness of phenotypes of symptomatic airways obstruction in adults. J Allergy Clin Immunol.

[10] Hinks T.S., Zhou X., Staples K.J., Dimitrov B.D., Manta A., Petrossian T. (2015). Innate and adaptive T cells in asthmatic patients: relationship to severity and disease mechanisms. J Allergy Clin Immunol.

[11] Juniper E.F., O'Byrne P.M., Ferrie P.J., King D.R., Roberts J.N. (2000). Measuring asthma control. Clinic questionnaire or daily diary?. Am J Respir Crit Care Med.

[12] Juniper E.F., Guyatt G.H., Epstein R.S., Ferrie P.J., Jaeschke R., Hiller T.K. (1992). Evaluation of impairment of health related quality of life in asthma: development of a questionnaire for use in clinical trials. Thorax.

[13] Zigmond A.S., Snaith R.P. (1983). The hospital anxiety and depression scale. Acta Psychiatr Scand.

[14] Piccirillo J.F., Merritt M.G., Richards M.L. (2002). Psychometric and clinimetric validity of the 20-Item Sino-Nasal Outcome Test (SNOT-20). Otolaryngol Head Neck Surg.

[15] Ware J.E., Sherbourne C.D. (1992). The MOS 36-item short-form health survey (SF-36). I. Conceptual framework and item selection. Med Care.

[16] Doty R.L., Shaman P., Kimmelman C.P., Dann M.S. (1984). University of Pennsylvania Smell Identification Test: a rapid quantitative olfactory function test for the clinic. Laryngoscope.

[17] Bafadhel M., McCormick M., Saha S., McKenna S., Shelley M., Hargadon B. (2012). Profiling of sputum inflammatory mediators in asthma and chronic obstructive pulmonary disease. Respiration.

[18] Carlsson G. (2009). Topology and data. Bull Am Math Soc.

[19] Lum P.Y., Singh G., Lehman A., Ishkanov T., Vejdemo-Johansson M., Alagappan M. (2013). Extracting insights from the shape of complex data using topology. Sci Rep.

[20] Chen W., Tabata Y., Gibson A.M., Daines M.O., Warrier M.R., Wills-Karp M. (2008). Matrix metalloproteinase 8 contributes to solubilization of IL-13 receptor alpha2 in vivo. J Allergy Clin Immunol.

[21] Monteseirin J., Vega A., Chacon P., Camacho M.J., El Bekay R., Asturias J.A. (2007). Neutrophils as a novel source of eosinophil cationic protein in IgE-mediated processes. J Immunol.

[22] Kita H., Abu-Ghazaleh R.I., Sur S., Gleich G.J. (1995). Eosinophil major basic protein induces degranulation and IL-8 production by human eosinophils. J Immunol.

[23] Moons K.G., Altman D.G., Reitsma J.B., Ioannidis J.P., Macaskill P., Steyerberg E.W. (2015). Transparent Reporting of a multivariable prediction model for Individual Prognosis or Diagnosis (TRIPOD): explanation and elaboration. Ann Intern Med.

[24] Moore W.C., Meyers D.A., Wenzel S.E., Teague W.G., Li H., Li X. (2010). Identification of asthma phenotypes using cluster analysis in the Severe Asthma Research Program. Am J Respir Crit Care Med.

[25] Simpson J.L., Scott R., Boyle M.J., Gibson P.G. (2006). Inflammatory subtypes in asthma: assessment and identification using induced sputum. Respirology.

[26] Kang M.J., Yoon C.M., Nam M., Kim D.H., Choi J.M., Lee C.G. (2015). Role of chitinase 3-like-1 in IL-18-induced pulmonary type-1, -2 and -17 inflammation, alveolar destruction and airway fibrosis in the murine lung. Am J Respir Cell Mol Biol.

[27] Ober C., Tan Z., Sun Y., Possick J.D., Pan L., Nicolae R. (2008). Effect of variation in CHI3L1 on serum YKL-40 level, risk of asthma, and lung function. N Engl J Med.

[28] Gomez J.L., Crisafi G.M., Holm C.T., Meyers D.A., Hawkins G.A., Bleecker E.R. (2015). Genetic variation in chitinase 3-like 1 (CHI3L1) contributes to asthma severity and airway expression of YKL-40. J Allergy Clin Immunol.

[29] Chupp G.L., Lee C.G., Jarjour N., Shim Y.M., Holm C.T., He S. (2007). A chitinase-like protein in the lung and circulation of patients with severe asthma. N Engl J Med.

[30] Otsuka K., Matsumoto H., Niimi A., Muro S., Ito I., Takeda T. (2012). Sputum YKL-40 levels and pathophysiology of asthma and chronic obstructive pulmonary disease. Respiration.

[31] Tang H., Sun Y., Shi Z., Huang H., Fang Z., Chen J. (2013). YKL-40 induces IL-8 expression from bronchial epithelium via MAPK (JNK and ERK) and NF-kappaB pathways, causing bronchial smooth muscle proliferation and migration. J Immunol.

[32] Mukhopadhyay S., Sypek J., Tavendale R., Gartner U., Winter J., Li W. (2010). Matrix metalloproteinase-12 is a therapeutic target for asthma in children and young adults. J Allergy Clin Immunol.

[33] Hunninghake G.M., Cho M.H., Tesfaigzi Y., Soto-Quiros M.E., Avila L., Lasky-Su J. (2009). MMP12, lung function, and COPD in high-risk populations. N Engl J Med.

[34] D'Armiento J., Dalal S.S., Okada Y., Berg R.A., Chada K. (1992). Collagenase expression in the lungs of transgenic mice causes pulmonary emphysema. Cell.

[35] Shapiro S.D. (2000). Animal models for chronic obstructive pulmonary disease: age of klotho and marlboro mice. Am J Respir Cell Mol Biol.

[36] Hautamaki R.D., Kobayashi D.K., Senior R.M., Shapiro S.D. (1997). Requirement for macrophage elastase for cigarette smoke-induced emphysema in mice. Science.

[37] Lemjabbar H., Gosset P., Lamblin C., Tillie I., Hartmann D., Wallaert B. (1999). Contribution of 92 kDa gelatinase/type IV collagenase in bronchial inflammation during status asthmaticus. Am J Respir Crit Care Med.

[38] Chaudhuri R., McSharry C., Brady J., Donnelly I., Grierson C., McGuinness S. (2012). Sputum matrix metalloproteinase-12 in patients with chronic obstructive pulmonary disease and asthma: relationship to disease severity. J Allergy Clin Immunol.

