# Supplementary material for: Multidimensional endotyping in patients with severe asthma reveals inflammatory heterogeneity in matrix metalloproteinases and chitinase 3–like protein 1
Source: J Allergy Clin Immunol. 2016 Jul;138(1):61–75. doi: 10.1016/j.jaci.2015.11.020 (PMC4929135; doi:10.1016/j.jaci.2015.11.020)
Supplement: Online Repository Data [file mmc1.doc]

**SUPPLEMENTARY APPENDIX**

Table of Contents

Supplementary information on methods 3

Inclusion criteria 3

Sample processing 3

Topological data analysis 4

Bayesian network analysis 5

Supplementary Results 5

Figure Legends 8

Figure E1. Flow diagram showing study design and recruitment. GINA, Global Initiative for Asthma; GORD, gastro-esophageal reflux disease; TDA, topological data analysis. 8

Figure E2. Multi-dimensional clinico-pathological clusters in asthma in validation dataset (Portsmouth cohort). 8

Figure E3. Analyses of matrix metalloproteinase / TIMP1 ratios generated from the clusters defined by the TDA network in Fig 2. 8

Figure E4. Analyses of mediators associated with type-2 inflammation. 9

Figure E5. Comparison IL17 cohort and Wessex Severe Asthma Cohorts. 9

Figure E6. Expression of sputum inflammatory mediators in eosinophilic asthma, neutrophilic asthma and health. 9

Figure E7. Correlates of neutrophilic inflammation. 10

Figure E8. Matrix metalloproteinases (MMP)s are positively correlated with markers of neutrophilic inflammation. 10

Figure E9. Matrix metalloproteinases are negatively correlated with interleukin (IL)-13. 10

Figure E10. Sputum IL-5 correlates more closely than IL13 with matrix metalloproteinases (MMP)s. 10

Figure E11. Correlates of airway eosinophilia. 10

Figure E12. Correlates of obesity. 11

Figure E13. Inflammatory mediators associated with sputum ECP. 11

Figure E14. Inflammatory mediators associated with sputum IL-5. 11

Figure E15. Inflammatory mediators associated with sputum IL-13. 11

Figure E16. Key parameters stratified according to GINA treatment group. 12

References 13

## Supplementary information on methods

### Inclusion criteria

Severe asthmatics comprised adult patients aged 18 to 70 years with severe asthma defined as difficult to control disease fulfilling the following criteria: 1) maintenance treatment at British Thoracic Society (BTS) asthma management steps 4 or 5, 2) persistent symptoms that required the need for short acting β_2_-agonist rescue medication for symptom relief and 3) a history of disease exacerbation within the preceding year that required a course of oral steroids or an increase in dose of maintenance oral steroids to achieve disease control and in whom alternative causes for symptoms had been excluded and in whom compliance with maintenance therapy is generally good^1^.

Mild asthmatics were selected on the basis of physician diagnosed asthma, abnormal bronchial hyperresponsiveness (BHR), as identified by abnormal PC_20_ methacholine value, and the need for prn β_2_-agonist use with or without low dose inhaled steroids (beclometasone or equivalent at ≤500μg/day) as their sole therapy.

Healthy controls had no current or previous symptoms to suggest asthma, normal lung function and normal BHR.

Exclusion criteria: asthmatics or healthy subjects with >10 pack-year cigarette smoking history; inter-current infection within 6 weeks before or 1 week after biological sampling; primary respiratory diagnosis other than asthma.

### Sample processing

Serum samples were coagulated for 30-60 minutes, centrifuged for 15 minutes at 1500g at 4°C, the serum layer removed and stored at -80°C until analysis.

Sputum induction was performed according to the recommendations of the European Respiratory Society using 7% hypertonic saline^2^ and the samples were immediately processed with 8x volume of phosphate buffered saline (PBS) for 30 minutes at 4°C then centrifuged and resuspended in 0.1% dithioerythritol (DTE) for 30 minutes to separate cells from the fluid phase of the sputum, as previously described.^3,4^ Cytospins were analyzed by rapid Romanowski staining.

Inflammatory mediators were measured by enzyme-linked immunosorbent assays (ELISA) – myeloperoxidase, elastase (Hycult), eosinophil cationic protein (MBL International), interleukin (IL)-5 (Abnova), IL-6, high-sensitivity-CRP (hs-CRP), ENA-78, Eotaxin, FGF, osteopontin, ST2/IL-1 R4, VEGF, YKL-40 (R&D Systems), Periostin (K Izuhara), tryptase and α2-macroglobulin (A Walls^4^) – by fluoroenzyme immunoassay – total and specific IgE (ImmunoCAP, Phadia)^5^ – or cytokine bead array (Luminex®): matrix metalloproteases (MMPs), metallopeptidase inhibitor 1 (TIMP-1), G-CSF, GM-CSF, Gro-α, CCL1, ICAM-1, IFN-γ, IL-1 α, IL-1 β, IL-1RA, IL-2, IL-4, IL-8, IL-10, IL-12p70, IL-13, IL-17, MCP-1, MIP-1 α, MIP-1 β, TNF-α. Urinary cotinine was measured by lateral flow chromatographic immunoassay (‘One Step’).

### Topological data analysis

A topological data analysis (TDA) constructed network represents a highly dimensional Venn diagram of related groups based on input column properties by binning several data points into single nodes and connecting edges amongst shared data points. TDA was performed using variance normalized Euclidean distance as a distance-metric. The Normalized Euclidean distance between two points takes into account that each column in the data set could have significantly different variance.

Twenty-nine parameters used to generate the TDA network are described in table E1, besides 74 parameters subsequently investigated but not used to define the groups.

### Bayesian network analysis

Seventy-four parameters were used in the Bayesian network analysis (BNA), and are described in table E2. Forty-one parameters were ultimately included by the model, whilst thirty-three were excluded from the model by the analysis.

## Supplementary Results

The study design and recruitment for each of the participant cohorts are described in figure E1. By definition mild asthma was defined as step 1 on the Global Initiative for Asthma (GINA) treatment algorithm, moderate as step 2, and severe as step 4 or 5 with persistent symptoms and a history of exacerbations.

The topological network generated from the validation cohort is shown in figure E2, with annotations statistically showing features which replicate features identified in the derivation cohort. Group i was did not have an identifiable counterpart in the derivation cohort.

The distributions of MMPs associated with asthma in this study are shown according to the TDA-derived clusters in Figure E3. There are elevated concentrations of at least one of these MMPs in each of the severe clusters B-H.

The distribution of markers associated with type-2 inflammation, serum periostin, sputum IL-5 and sputum IL-13 are shown in figure E4. Serum perisotin was normal or elevated in clusters B,E,F and significantly reduced in clusters C and H. As in a previous study^4^, we observed a reciprocal relationship between sputum IL-5 and IL-13, likely related to steroids. Sputum IL-5 is elevated in severe asthma, significantly so in clusters B,E,F,H, whilst sputum IL-13 is reduced in each of the severe clusters, significantly so in clusters B, F, H.

Table E3 and figure E5 present a qualitative comparison of the clusters identified in this study and those identified in the separate ‘IL-17 cohort’ previously described^4^.

Figure E6 presents data on sputum inflammatory mediators when subjects have been stratified according to eosinophilic or neutrophilic subtype (other asthmatic subjects are excluded). Sputum YKL-40 is significantly more elevated in neutrophilic than eosinophilic asthma, whilst eosinophil cationic protein (ECP) and IL-5 are siginificantly more elevated in eosinophilic asthma. In contrast to IL-5, another type-2 cytokine IL-13 is significantly reduced compared with health in severe neutrophilic or severe eosinophilic asthma.

The associations observed in the Bayesian network analysis (Figure 3) are shown in greater detail using univariate Spearman’s correlations in figures E7 to E12. Sputum myeloperoxidase (released by airway neutrophils) is strongly correlated with sputum ECP, elastase and IL-8, and moderately correlated with the pro-inflammatory cytokine IL-1β (figure E7). Sputum MMP12 is highly connected within the Bayesian network, and thus has moderately strong correlations with MMPs-1, -3 and -8 (the three secreted type 3 collagenases) besides the markers of neutrophilic inflammation sputum neutrophil counts, elastase, YKL-40 and myeloperoxidase (MPO)(Figure E8).

In contrast to many positive correlations, several MMPs are significantly negatively correlated with sputum IL-13 (figure E9). Conversely sputum IL-5 is positively correlated with these MMPs (figure E10) providing further evidence of a complex relationship between IL-5 and IL-13. As expected from the Bayesian network IL-5, sputum eosinophils and sputum ECP are all positively correlated (Figure E11).

We found confirmatory evidence of other groups’ observations^6^ an overlap between obesity, neutrophilia and hs-CRP, consistent with systemic inflammation (Figure E12).

In observational studies of severe asthma it is hard to correct for the confounding effect of therapeutic corticosteroids possibly generating observed differences. It would be technically very challenging to incorporate an algorithm to correct for steroid use in the TDA or BNA. However figure E16 presents twelve sputum parameters – representing key observations in this study – stratified according to GINA treatment group. In addition to one-way ANOVA with *post hoc* Dunnett’s comparisons between each group and health (shown in figure) we also compared distributions for GINA step 4 (receiving a median dose of 2000 mcg BDP equivalent) with participants on step 5 (receiving the same median dose of ICS but also an additional median 10mg/day oral prednisone). For each of these twelve parameters, no significant differences were observed between GINA steps 4 and 5, which would argue against our main observations being attributable wholly to therapeutic corticosteroids.

## Figure Legends

### Figure E1. Flow diagram showing study design and recruitment. GINA, Global Initiative for Asthma; GORD, gastro-esophageal reflux disease; TDA, topological data analysis.

### Figure E2. Multi-dimensional clinico-pathological clusters in asthma in validation dataset (Portsmouth cohort).

Topological network generated using 22 clinical and pathological features together identifies one healthy (in blue) and eight distinct clinico-pathobiological asthma clusters (A-H). The network is colored according to ACQ7 score with the most symptomatic subjects in red.

The TDA used 70 subjects with most complete data; Metric: variance normalized Euclidean; Lenses: principal and secondary singular value decomposition (SVD)(Resolution 30, Gain 3.0/4.0x, Equalized) and presence/absence of asthma; Node size: proportional to number of individuals in node. Color bars: red, highest ACQ7 score; blue, healthy participants. Features in bold typeface were replicated in the validation dataset.

### Figure E3. Analyses of matrix metalloproteinase / TIMP1 ratios generated from the clusters defined by the TDA network in Fig 2.

Ratios of concentrations of MMPs -1, -3, -8, and -12 to TIMP1 in induced sputum in data from derivation (Southampton, panels A-D) and validation (Portsmouth, panels E-H) cohorts. Data shown only for clusters which were replicated in both cohorts. Scatter plots of log-transformed data, with means. Statistical tests indicated 1-way ANOVA with post hoc Dunnett’s tests compared with healthy subjects. *P <.05, ** P<.01, *** P<.001, **** P<.0001. HC, healthy controls.

### Figure E4. Analyses of mediators associated with type-2 inflammation.

Plots generated from the clusters defined by the TDA network in Fig 2 show concentrations of periostin in serum, and interleukin-5 and -13 in induced sputum from derivation (Southampton, panels A-C) and validation (Portsmouth, panels D-F) cohorts. Data shown only for clusters which were replicated in both cohorts. Scatter plots of log-transformed data, with means. Statistical tests indicated 1-way ANOVA with post hoc Dunnett’s tests compared with healthy subjects. *P <.05, ** P<.01, *** P<.001, **** P<.0001. HC, healthy controls.

### Figure E5. Comparison IL17 cohort and Wessex Severe Asthma Cohorts.

Qualitative comparison of the multidimensional clusters identified by TDA in the Wessex Severe Asthma Cohort (WSAC) (right) with those identified in the previous study using similar methodology (‘IL17 cohort’^4^) (left). Broken lines link clusters with similar properties in each study.

### Figure E6. Expression of sputum inflammatory mediators in eosinophilic asthma, neutrophilic asthma and health.

(A) sputum YKL40 (chitinase-3-like protein 1), (B) serum eosinophil cationic protein (ECP), (C) sputum interleukin (IL)-5, (D) sputum IL-13. Scatterplots with medians. Statistical comparisons by 1-way ANOVA with post hoc Tukey’s tests on log-transformed data. *P <.05, ** P<.01, *** P<.001, **** P<.0001.

### Figure E7. Correlates of neutrophilic inflammation.

Spearman’s correlations between sputum myeloperoxidase (MPO) and eosinophil cationic protein (ECP) (A), elastase (B), interleukin (IL) -1β (C), interleukin-8 (D). rs, Spearman’s correlation coefficient.

### Figure E8. Matrix metalloproteinases (MMP)s are positively correlated with markers of neutrophilic inflammation.

Spearman’s correlations between different MMPs and inflammatory cells or their mediators in induced sputum. rs, Spearman’s correlation coefficient.

### Figure E9. Matrix metalloproteinases are negatively correlated with interleukin (IL)-13.

Spearman’s correlations between IL-13 and matrix metalloproteinase (MMP) / TIMP-1 ratios for (A) MMP3, (B) MMP8, (C) MMP9, (D) MMP12 in induced sputum. rs, Spearman’s correlation coefficient.

### Figure E10. Sputum IL-5 correlates more closely than IL13 with matrix metalloproteinases (MMP)s.

Spearman’s correlations between mediators in induced sputum. rs, Spearman’s correlation coefficient.

### Figure E11. Correlates of airway eosinophilia.

Spearman’s correlations in induced sputum between (A) interleukin (IL)-5 and eosinophil cationic protein (ECP), (B) eosinophils and IL-5, (C) eosinophils and ECP. rs, Spearman’s correlation coefficient.

### Figure E12. Correlates of obesity.

Spearman’s correlations between body mass index (BMI) and (A) high sensitivity C reactive protein (hs-CRP) and (B) log blood neutrophil count. rs, Spearman’s correlation coefficient.

### Figure E13. Inflammatory mediators associated with sputum ECP.

Spearman’s correlations between sputum ECP and sputum YKL-40 (A), IL-8 (B), MIP1B (C), MMP3 (D), IL-6SR (E), VEGF (F), MIP1A (G), MMP8 (H).

ECP, eosinophil cationic protein; IL, interleukin; MIP, macrophage inflammatory protein; MMP, matrix metalloproteinase; rs, Spearman’s correlation coefficient; SR, soluble receptor; YKL-40, chitinase-3-like protein 1; VEGF, vascular endothelial growth factor.

### Figure E14. Inflammatory mediators associated with sputum IL-5.

Spearman’s correlations between sputum IL-5 and sputum YKL-40 (A), IL-8 (B), MIP1B (C), MMP3 (D), IL-6SR (E), VEGF (F), MIP1A (G), MMP8 (H).

ECP, eosinophil cationic protein; IL, interleukin; MIP, macrophage inflammatory protein; MMP, matrix metalloproteinase; rs, Spearman’s correlation coefficient; SR, soluble receptor; YKL-40, chitinase-3-like protein 1; VEGF, vascular endothelial growth factor.

### Figure E15. Inflammatory mediators associated with sputum IL-13.

Spearman’s correlations between sputum IL-13 and sputum YKL-40 (A), IL-8 (B), MIP1B (C), MMP3 (D), eotaxin (E), tryptase (F), ECP(G), MMP12 (H).

ECP, eosinophil cationic protein; IL, interleukin; MIP, macrophage inflammatory protein; MMP, matrix metalloproteinase; *r*_s_, Spearman’s correlation coefficient; YKL-40, chitinase-3-like protein 1.

### Figure E16. Key parameters stratified according to GINA treatment group.

Sputum parameters of specific interest in this paper are presented according to GINA treatment group. Group 1 is steroid-naïve, group 2 low dose ICS (median 400 mcg/BDP), group 4 high dose ICS (median 2000mcg/BDP), group 5 high dose ICS (median 2000 mcg/BDP) with long term oral corticosteroids (median 10mg/day prednisolone). P values are for comparisons of groups using one-way ANOVA on log transformed data with *post-hoc* Dunnett’s test compared with HC. *P<0.05, **P<0.01. ***P<0.001, ****P<0.0001. Additionally no significant differences were observed between groups 4 and 5 for any of these parameters using unpaired t tests on log-transformed data. MMP-1 (A), MMP-3 (B), MMP-8 (C), MMP-12 (D), VEGF (E), YKL-40 (F). neutrophil count (G), MPO (H), IL-8 (I), IL-6 (J), IL-6SR (K), elastase (L).

BDP, beclometasone dipropionate equivalent; GINA, global initiative for asthma; HC, healthy control; ICS, inhaled corticosteroid; IL, interleukin; MMP, matrix metalloproteinase; MPO, myeloperoxidase; SR, soluble receptor; VEGF, vascular endothelial growth factor; YKL-40, chitinase-3-like protein 1.

## References

1. Holgate ST, Polosa R. The mechanisms, diagnosis, and management of severe asthma in adults. Lancet 2006;368:780-93.

2. Djukanovic R, Sterk PJ, Fahy JV, Hargreave FE. Standardised methodology of sputum induction and processing. Eur Respir J Suppl 2002;37:1s-2s.

3. Staples KJ, Hinks TS, Ward JA, Gunn V, Smith C, Djukanovic R. Phenotypic characterization of lung macrophages in asthmatic patients: Overexpression of CCL17. J Allergy Clin Immunol 2012;130:1404-12 e7.

4. Hinks TS, Zhou X, Staples KJ, et al. Innate and adaptive T cells in asthmatic patients: Relationship to severity and disease mechanisms. J Allergy Clin Immunol 2015.

5. Bachert C, van Steen K, Zhang N, et al. Specific IgE against Staphylococcus aureus enterotoxins: an independent risk factor for asthma. J Allergy Clin Immunol 2012;130:376-81 e8.

6. Fingleton J, Travers J, Williams M, et al. Treatment responsiveness of phenotypes of symptomatic airways obstruction in adults. J Allergy Clin Immunol 2015.
