# Supplementary material for: Multidimensional endotyping in patients with severe asthma reveals inflammatory heterogeneity in matrix metalloproteinases and chitinase 3–like protein 1
Source: J Allergy Clin Immunol. 2016 Jul;138(1):61–75. doi: 10.1016/j.jaci.2015.11.020 (PMC4929135; doi:10.1016/j.jaci.2015.11.020)
Supplement: Tables E1-E3 [file mmc2.doc]

### Table E1. Parameters used in the topological data analysis

| **Twenty-nine parameters used to generate the TDA network** | | | |  |  |
| --- | --- | --- | --- | --- | --- |
|  | **Demographic** | |  |  |  |
|  |  | Presence of asthma | Gender | Age | Age at Diagnosis |
|  |  | Whether developed asthma after age 12 |  |  |  |
|  | **Pulmonary function** | |  |  |  |
|  |  | Reversibility | FEV_1_ (% predicted, post BD) | Exhaled nitric oxide |  |
|  | **Clinical parameters** | |  |  |  |
|  |  | Step on GINA treatment algorithm | Atopic status | Number of SPT responses | Total serum IgE |
|  |  | History of symptoms induced by aspirin | Body mass index | Current smoking status | History of smoking (pack years) |
|  |  | ACQ6 | Rhinosinusitis | AQLQ score | Hyposmia |
|  |  | SNOT-20 |  |  |  |
|  | **Peripheral blood** | |  |  |  |
|  |  | Peripheral blood eosinophil count | Peripheral blood neutrophil count | Serum high-sensitivity CRP | Serum periostin |
|  | **Sputum inflammation** | |  |  |  |
|  |  | Sputum neutrophil count | Sputum eosinophil count | Neutropilic sputum subclass^A^ | Eosinophilic sputum subclass^B^ |
|  |  |  |  |  |  |
| **Seventy-four parameters investigated, but not used to generate the TDA network** | | | |  |  |
|  | **Demographic** | |  |  |  |
|  |  | Disease Duration | Whether developed asthma after age 18 |  |  |
|  | **Pulmonary function** | |  |  |  |
|  |  | FEV_1_/FVC ratio | FEV_1_ (% predicted, pre BD) |  |  |
|  | **Clinical parameters** | |  |  |  |
|  |  | Inhaled corticosteroid dose | ACQ7 | AQLQ: Activity component | AQLQ: Emotional component |
|  |  | AQLQ; Environmental component | AQLQ: Symptom component | HADS (Hospital Anxiety and depression scale) | HAD: Anxiety component |
|  |  | HAD: Depression component | SF-36 (Short Form (36) Health Survey) | Urinary cotinine test | Staph Endotoxin-specific IgE |
|  |  | House dust mite sensitivity (SPT) | Cat sensitivity (SPT) | Perennial aeroallergen sensitivity (SPT) | SPT allergen wheal size |
|  |  | Nasal Polyposis | Olfaction Score | Nasal dysfunction score^C^ |  |
|  | **Peripheral blood** | |  |  |  |
|  |  | Serum interleukin-6 | Serum ST2L (IL-1 R4) | Serum YKL40 |  |
|  | **Sputum inflammation** | |  |  |  |
|  |  | Sputum lymphocyte count | Sputum fibroblast growth factor | Sputum IL-1α | Sputum myeloperoxidase (MPO) |
|  |  | Sputum macrophage count | Sputum α-2 macroglobulin | Sputum IL-1β | Sputum osteopontin (OPN) |
|  |  | Sputum epithelial cell count | Sputum eosinophil cationic protein | Sputum IL-1RA | Sputum TIMP-1 |
|  |  | Sputum squamous cell count | Sputum Elastase | Sputum IL-2 | Sputum TNF-α |
|  |  | Sputum quality (whether <40% squamous contamination) | Sputum ENA78 | Sputum IL-4 | Sputum Tryptase |
|  |  | Mixed granulocytic sputum subclass | Sputum Eotaxin | Sputum IL-5 | Sputum VEGF |
|  |  | Paucigranulocytic sputum subclass | Sputum G-CSF | Sputum IL-6 | Sputum YKL40 |
|  |  | Overall sputum subclass | Sputum GM-CSF | Sputum IL-6 soluble receptor | Sputum MMP1/TIMP-1 ratio |
|  |  |  | Sputum Gro-α (CXCL1) | Sputum IL-8 | Sputum MMP2/TIMP-1 ratio |
|  |  |  | Sputum IFN-γ | Sputum IL-10 | Sputum MMP3/TIMP-1 ratio |
|  |  |  | Sputum MCP1 | Sputum IL-13 | Sputum MMP7/TIMP-1 ratio |
|  |  |  | Sputum MIP-1α | Sputum IL-17 | Sputum MMP8/TIMP-1 ratio |
|  |  |  | Sputum MIP-1β |  | Sputum MMP9/TIMP-1 ratio |
|  |  |  |  |  | Sputum MMP12/TIMP-1 ratio |
|  |  |  |  |  | Sputum MMP13/TIMP-1 ratio |

A. Sputum neutrophil ≥61%. B. Sputum eosinophil count ≥3%. C. Composite average of hyposmia, rhinosinusitis and SNOT-20 score.

ACQ6, 6-point Asthma Control Questionnaire; AQLQ, Asthma Quality of Life Questionnaire; BD, Bronchodilator; BTS, British Thoracic Society; CRP, C-reactive protein; FVC, Forced Vital Capacity; FEV_1_, Forced Expiratory Volume in 1 second; GINA, Global Initiative for Asthma; SNOT-20, 20-point Sino-Nasal Outcomes Test; SPT, skin prick testing.

### Table E2. Parameters used in the Bayesian network analysis

| **Forty-one parameters included in the model by the analysis** | | | |  |  |
| --- | --- | --- | --- | --- | --- |
|  | **Demographic** | |  |  |  |
|  |  | Age at Diagnosis |  |  |  |
|  | **Pulmonary function** | |  |  |  |
|  |  | FEV_1_ (% predicted, post BD) |  |  |  |
|  | **Clinical parameters** | |  |  |  |
|  |  | Step on GINA treatment algorithm | Atopic status | Number of SPT responses | Inhaled corticosteroid dose |
|  |  | Disease Duration | HADS (Hospital Anxiety and depression scale) | SF-36 (Short Form (36) Health Survey) | Sinonasl symptoms (SNOT-20) |
|  |  | ACQ7 | AQLQ score |  |  |
|  | **Sputum inflammation** | |  |  |  |
|  |  | Sputum neutrophil count | Sputum VEGF | Sputum IL-2 | Sputum MIP-1α |
|  |  | Sputum eosinophil count | Sputum FGF | Sputum IL-4 | Sputum MIP-1β |
|  |  | Sputum macrophage count | Sputum G-CSF | Sputum IL-5 | Sputum TNF-α |
|  |  | Sputum myeloperoxidase | Sputum YKL40 | Sputum IL-6 soluble receptor | Sputum MMP1/TIMP-1 ratio |
|  |  | Sputum Elastase | Sputum IL-1α | Sputum IL-8 | Sputum MMP12/TIMP-1 ratio |
|  |  | Sputum eosinophil cationic protein | Sputum IL-1β | Sputum IL-10 | Sputum MMP13/TIMP-1 ratio |
|  |  |  | Sputum IL-1RA | Sputum IL-13 | Sputum MMP3/TIMP-1 ratio |
|  |  |  |  | Sputum IL-17 | Sputum MMP8/TIMP-1 ratio |
| **Thirty-three parameters excluded from the model by the analysis** | | | |  |  |
|  | **Demographic** | |  |  |  |
|  |  | Gender |  |  |  |
|  | **Pulmonary function** | |  |  |  |
|  |  | Exhaled nitric oxide | Reversibility |  |  |
|  | **Clinical parameters** | |  |  |  |
|  |  | Body mass index | History of symptoms induced by aspirin | Olfaction score | Nasal polyposis |
|  |  | Current smoking status |  |  |  |
|  | **Peripheral blood** | |  |  |  |
|  |  | Peripheral blood eosinophil count | Total serum IgE | Serum high sensitivity CRP | Serum ST2L (IL-1 R4) |
|  |  | Peripheral blood neutrophil count | Staph Endotoxin-specific IgE | Serum Periostin | Serum YKL40 |
|  | **Sputum inflammation** | |  |  |  |
|  |  | Sputum lymphocyte count | Sputum GM-CSF | Sputum ENA78 | Sputum MMP2/TIMP-1 ratio |
|  |  | Sputum lymphocyte count | Sputum MCP1 | Sputum Gro-α (CXCL1) | Sputum MMP7/TIMP-1 ratio |
|  |  | Sputum epithelial cell count | Sputum eotaxin | Sputum α-2 macroglobulin | Sputum MMP9/TIMP-1 ratio |
|  |  | Sputum eosinophil cationic protein | Sputum tryptase | Sputum IFN-γ |  |
|  |  |  | Sputum osteopontin | Sputum IL-6 |  |

A. Sputum neutrophil ≥61%. B. Sputum eosinophil count ≥3%. C. Composite average of hyposmia, rhinosinusitis and SNOT-20 score.

ACQ6, 6-point Asthma Control Questionnaire; AQLQ, Asthma Quality of Life Questionnaire; BD, Bronchodilator; BTS, British Thoracic Society; CRP, C-reactive protein; FVC, Forced Vital Capacity; FEV_1_, Forced Expiratory Volume in 1 second; GINA, Global Initiative for Asthma; SNOT-20, 20-point Sino-Nasal Outcomes Test; SPT, skin prick testing.

### Table E3. Comparison of clusters with multidimensional endotypes described in Hinks, Zhou *et al* 2015

| **IL17 cohort** | | **Wessex severe asthma cohort** | |
| --- | --- | --- | --- |
| Hinks *TS et al J Allergy Clin Immunol 2015* | |  |  |
| **Cluster** | **Features of cluster** | **Cluster** | **Features of cluster** |
| 1 | Mild asthma symptoms | A | Mild asthma symptoms |
|  | Pauci-granulocytic sputum |  | Pauci-granulocytic sputum |
|  | More likely GINA 1-2 |  | More likely GINA 1-2 |
|  | Young |  | Young |
|  | Th2-high |  | Low periostin, high serum ECP |
| 2 | Well-controlled asthma | N/A | Not replicated in the present study^B^ |
|  | Eosinophilic |  |  |
| 3 | Moderately severe asthma | N/A | Not replicated in the present study^B^ |
|  | Moderately High eNO, Th2-/Th17-high |  |  |
|  | Th2-/Th17-high |  |  |
| 4 | Moderately-severe airflow obstruction and symptoms | F | Severe airflow obstruction and symptoms |
|  |  |  |  |
|  | Nasal polyposis |  | Nasal polyposis |
|  | Salicylate-sensitive |  | Salicylate-sensitive disease |
|  |  |  | High periostin, high sputum eosinophils and neutrophils, depression, high sputum ECP |
|  |  |  |  |
| 5 | Severe symptoms | E | Severe symptoms |
|  | Atopic |  | Atopic |
|  | Older (median age 52) |  | Median age 45 |
|  | Early onset (median age 5) |  | Early onset (median age 4) |
|  | High type-2 cytokines |  | High sputum IL5 |
|  | High mast cell mediators, obese |  | Eosinophilic disease, high periostin, high FeNO. |
| 6 | Severe symptoms, neutrophilic, highest ACQ (median 3.4), severe airflow obstruction, frequently (43%) oral corticosteroids, obese, non-atopic, predominantly female, high mast cell mediators | H | Severe symptoms, neutrophilic disease, highest ACQ (median 3.3), severe airflow obstruction, frequently (33%) oral corticosteroids, low periostin |
|  |  |  |  |

A. Eight subjects participated in both these studies. Data were obtained under distinct protocols, under separate ethics, at different visits. B. Well-controlled moderately severe asthmatics are under-represented in the Wessex severe asthma cohort.
